# Systematic review to evaluate a potential association between helminth infection and physical stunting in children

**DOI:** 10.1186/s13071-022-05235-5

**Published:** 2022-04-20

**Authors:** E. Raj, B. Calvo-Urbano, C. Heffernan, J. Halder, J. P. Webster

**Affiliations:** 1 Department of Pathobiology and Population Sciences, Royal Veterinary College, University of London, Hertfordshire, AL9 7TA UK; 2 London Centre for Neglected Tropical Disease Research, Imperial College Faculty of Medicine, W2 1PG London, UK; 3 London International Development Centre, London, WC1A 2NS UK

**Keywords:** Soil-transmitted helminths, Schistosomes, Stunting, Children, Infants, Pregnant women, Systematic review, Meta-analyses, z-scores

## Abstract

**Background:**

Despite considerable public health efforts over the past 20 years, childhood stunting (physical and/or cognitive) levels globally remain unacceptably high—at 22% amongst children under 5 years old in 2020. The aetiology of stunting is complex and still largely unknown. Helminths can cause significant mortality and morbidity and have often been cited as major causative agents for stunting, although their actual role in childhood stunting remains unclear. Our aim was to systematically review the current evidence to help support or refute the hypothesis that helminths cause physical stunting in children.

**Methods:**

Inclusion criteria were as follows: infected with (and/or exposed to) helminths (soil-transmitted helminths, schistosomes or food-borne trematodes), children, pregnant or breastfeeding women as study participants (children included infants 0–1 year old, preschool-age children 1–5 years and school-age children > 5 years old), anthelmintic treatment intervention, stunting-related variables reported (e.g. height, height-for-age z-score, birth weight), helminth infection reported in relation to stunting, any geographic location, any date, peer-reviewed literature only. Exclusion criteria were: non-primary research, study protocols, studies with no new data, non-English language papers and animal (non-human) helminth studies. Seven databases were searched on 28 May 2021. Risk of bias was assessed for included studies and GRADE was used for studies included in RCT subgroup meta-analyses (in preschool-age children and pregnant women). This systematic review was registered with PROSPERO (CRD42021256201).

**Results:**

Eighty studies were included in the analyses. No significant overall evidence was found in support of the hypothesis that helminths cause physical stunting in children, although there was some association with wasting.

**Conclusions:**

Whilst analyses of the available literature to date failed to support a direct association between helminth infection and childhood stunting, there was significant heterogeneity between studies, and many had follow-up periods which may have been too short to detect impacts on growth. Most apparent was a lack of available data from key demographic groups wherein one may predict the greatest association of helminth infection with stunting—notably that of infants, preschool-age children, and pregnant or nursing women. Thus this review highlights the urgent need for further targeted empirical research amongst these potentially most vulnerable demographic groups.

**Graphical Abstract:**

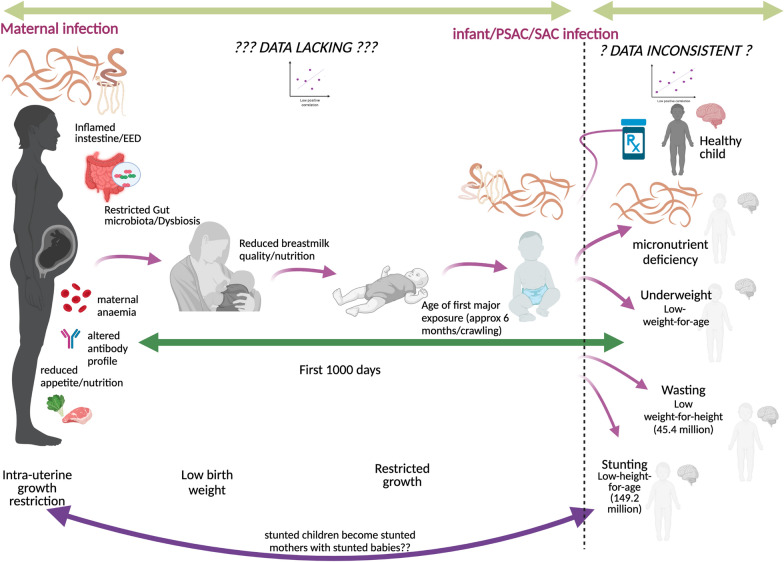

**Supplementary Information:**

The online version contains supplementary material available at 10.1186/s13071-022-05235-5.

## Background

Childhood stunting is a major public health issue leading to lifelong adverse health, education and economic outcomes [1, 2]. While public health efforts have led to a considerable reduction in the global number of stunted children over the past 20 years, from 203 to 149 million [3], stunting levels remain unacceptably high. Indeed, the absolute figure for stunted children in Central and West Africa has risen during this time, partly due to the speed of population growth [3]. Data show that 22% of all children under 5 years old were stunted in 2020 [3], despite specific targets such as those by the World Health Organization (WHO) to reduce stunting by 40% in children under 5 by 2025 [4].

Stunting refers to both reduced physical growth and cognitive impairment, although the WHO definition focuses on the anthropometric aspect, where stunted children have a height for age that is “more than 2 standard deviations below the WHO child growth standards median” [5]. However, children who are not stunted according to this definition can still be short for their age and suffer from growth faltering [2]. Stunting reflects chronic undernutrition [6] and is the most common form of malnutrition in children under 5 [1–3]. Other forms of undernutrition include wasting (low weight for height), underweight (low weight for age) and micronutrient deficiency [6].

Stunted children have higher morbidity and mortality risks compared with non-stunted children [7]. Cognitive stunting leads to poorer school performance and an inability to reach their full potential [7]. This has adverse economic consequences for future productivity and earnings [8]. Stunted children also suffer a double burden of malnutrition, as evidence shows they have an increased risk of non-communicable diseases in later life [9, 10]. The importance of childhood stunting globally is emphasised by its inclusion in the calculation of the Global Hunger Index [11], as well as the Global Nutrition Targets 2025 [4].

Multiple factors are thought to contribute to stunting, including, but not exclusive to, nutrition, infections, the environment and maternal health [7]. Stunting also has an intergenerational aspect, where women with short maternal stature are more likely to have stunted children [2, 12]. It can therefore be challenging to disentangle which aspects have the greatest effects and, hence, which elements should be prioritised for interventions and integrated into public health programmes. Stunting and helminthiasis are also strongly associated with poverty and affect the most vulnerable populations [7, 13–15]. Helminth infections (i.e. parasitic worms) have been proposed as a major cause of stunting [16]. Helminthiasis caused by soil-transmitted helminths (STHs), *Schistosoma* spp. and food-borne trematodes (FBTs) can lead to significant morbidity [17–19], ranging from anaemia, haematuria, hepatomegaly and portal hypertension to intestinal obstruction, rectal prolapse and cancer in severe cases. Most people in endemic regions suffer from multiple helminth species infections concurrently [20, 21]. Children are especially vulnerable to infection due to their hygiene and play habits [22]. A recent study [23] showed that preschool-age children (PSAC) with schistosomiasis had significantly poorer early childhood development scores, but that these improved to the same levels as un-infected children with anthelmintic treatment. Along with children, pregnant and breastfeeding women have also been suspected as potential high-risk groups for helminth infections [16], due to hormonal changes and possible immunosuppression [24]. WHO classifies stunting as a subtle morbidity of helminthiasis [16], yet the short-term and long-term adverse health and economic consequences of stunting are significant for affected individuals and wider communities [2].

The first 1000 days of a child’s life, from conception to their second birthday, are recognised as the most critical time for growth and development [1, 2]. Yet helminth-infected PSAC and pregnant and nursing women have only more recently been included, and indeed actively promoted as regards the latter, in treatment programmes [25]. For infants and PSAC with schistosomiasis, this can partly be explained by the lack of a suitable licensed praziquantel formulation [26], although a paediatric praziquantel is currently in phase III clinical trials [27]. For pregnant and nursing women, their initial exclusion was due to, now alleviated, lack of safety data regarding anthelmintic use in these groups [28–30].

Evidence relating to the often-cited association between helminth infections in children and physical stunting [7, 31–33] is, however, often conflicting. Some studies propose no association [34, 35], several report a positive association [36, 37], whilst others propose associations for specific helminth species only (such as *Ascaris lumbricoides* only) [38]. Intensity of infection is acknowledged to be a potential key factor, with associations sometimes only found for moderate or high-intensity infections [39], although others suggest a different dynamic, where stunting is a predictor of STH infection [40]. An updated Cochrane review concluded that deworming did “not appear to improve height, haemoglobin, cognition, school performance, or mortality” [41]. However, the Cochrane review focused on STHs only and randomised controlled trials (RCTs) or quasi-RCTs. Whilst Cochrane systematic reviews are considered the gold standard in human medicine, their rigid protocols may not always allow the necessary scope to explore multifactorial and complex concepts such as stunting. Other systematic reviews on the topic of helminths and stunting in children are also already available, although to date these have either examined different aspects—notably that of cognitive rather than physical stunting [42], a range of outcomes based on secondary data from demographic and health surveys [43], or sanitation [44], and/or were restricted to STHs only [45, 46].

Bearing in mind the current knowledge gaps, our study therefore aimed to systematically review the available literature and synthesise the evidence to evaluate the potential association between helminth infection and physical stunting in children, notably with an aim to uniquely include infants and PSAC in particular, as well as pregnant and nursing women. Furthermore, whilst most studies to date have focused on STHs or schistosomiasis, few have examined both, and to our knowledge, no systematic reviews on this topic to date have aimed to include *Strongyloides stercoralis* or the FBTs. Finally, our review aimed to cast a wider net than the Cochrane systematic review [41] as it incorporated a range of study designs, including case reports and case series, rather than only RCTs.

## Methods

This review followed the Preferred Reporting Items for Systematic Reviews and Meta-Analyses (PRISMA) guidelines [47, 48] and was registered with the International Prospective Register of Systematic Reviews (PROSPERO) on 28 June 2021. Registration number; CRD42021256201.

A search strategy was developed using the **PICO** approach, by breaking down the research question:

- **P**opulation and problem—infants and children, pregnant and breastfeeding women infected with (and/or exposed to) helminths
- **I**ntervention—treatment with an anthelmintic
- **C**omparison—treated vs controls, infected vs un-infected or pre- and post-treatment
- **O**utcome—physical stunting in children (a sub-set of the population of interest).

The search strategy comprised terms representing four concepts: helminths, stunting-related variables, the relevant populations (children, pregnant and breastfeeding women) and treatment. Relevant medical subject headings were also used as part of the search strategy for Medline, Embase and Global Health. Cognitive stunting terms were not included in the search strategy as this review focused on physical stunting. Since the term “children” covers a wide range of ages, for clarity, this review, and the search strategy therein, was selected from the age thresholds provided in WHO’s STH preventive chemotherapy guidelines as shown in Table 1 [16].

**Table 1:** Age group category thresholds for children (as defined by WHO [16])

Group	Current WHO guidelines	This review
	Age (months)	Age (years)
Infants	0 to 12	0 to 1
Young children	12+ to 23	1+ to 5
Preschool-age children (PSAC)	24 to 59	1+ to 5
School-age children (SAC)	5 to 14 years old	5+ to 20

Infants, young children and PSAC were grouped together here as “PSAC” (due to the age ranges covered by included studies), and our SAC grouping included a wider age range to accommodate the available studies. (Please see Supplementary information for study protocol [Additional file 1], search concepts [Additional file 2] and search strategies [Additional file 3]).

A total of seven databases were searched to identify studies. These were chosen based on the topics and regions covered. The databases searched were MEDLINE, Embase, Global Health, Africa-Wide Information, Latin American and Caribbean Health Sciences Literature (LILACS), Scopus and Web of Science.

Searches were carried out on 28 May 2021. No language, date limits or other filters were used at the search stage. Searches sought published peer-reviewed literature only. Search results from all seven databases were exported to EndNote reference manager (Clarivate Analytics) [49], de-duplicated and screened.

Titles and abstracts of search result records were initially screened using the inclusion and exclusion criteria shown in Table 2.

**Table 2:** Inclusion and exclusion criteria

Inclusion criteria	Exclusion criteria
Infection with (and/or exposure) to helminths—STHs, schistosomes or FBTs	Non-primary research—systematic reviews, commentaries and editorials
Participants—children, pregnant or breastfeeding women	Study protocols
Anthelmintic treatment intervention	Studies with no new or novel data
Stunting-related variables reported—height, HAZ, proportion stunted, birth weight	Non-English language papers
Reported outcomes—helminth infection in participants in relation to stunting	Animal (non-human) helminth studies
Any geographic location	
Any date	

Sources: peer-reviewed literature only

Data were collected in Excel [50] by one main reviewer, with another working independently to double-check that appropriate and correct data were extracted for potential meta-analysis.

Data were sought relating to prevalence of helminth infection (for example, infected vs un-infected), intensity of infection if present (such as low, moderate or heavy), diagnostics used (for example, Kato-Katz or urine filtration) and time until follow-up. Data were obtained from studies regarding stunting-related variables in children, in particular measurements of height, height/length-for-age z-scores (HAZ/LAZ), supine length, proportion stunted, low birth weight (LBW, < 2500 g) and very low birth weight (VLBW, < 1500 g). Information was also sought relating to the age of children, location, year of study and treatment intervention.

Studies were assessed for risk of bias by checking for randomisation, comparator groups, adequate blinding (multi-arm studies only), sensitivity and appropriateness of diagnostic tests and potential confounding. The Grading of Recommendations Assessment, Development and Evaluation (GRADE) framework [51] was used to assess the risk of bias at the outcome level for RCT studies included in subgroup meta-analyses. It was not possible to use GRADE to assess the quality and certainty of evidence for all included studies, since it relies on comparison groups (e.g. treatment and control groups) [52].

For height and birth weight, mean difference and standard deviation (SD) of the mean difference were used in the synthesis and presentation of results. For HAZ/LAZ, the mean difference and SD of the mean difference were used for synthesis of results, and for LBW and VLBW, odds ratios were used.

A total of 80 studies were included in this review, and results were initially synthesised narratively, grouped by study design.

### Meta-analyses

Meta-analyses were performed in R [53] using the “meta” package for two key subgroups: PSAC and pregnant women. Odds ratios were obtained for binary outcomes (e.g. LBW) and mean differences were calculated for continuous outcomes (e.g. height). Fixed and random-effects were employed for pooling the results of the individual analyses. The Mantel–Haenszel (MH) method was used for pooling the odds ratios (i.e. calculating weights), and the inverse variance method was employed in the case of continuous variables. The between-study variance *τ*^2^ in the random-effects model was estimated with the DerSimonian and Laird method. The *I*^2^ statistic was used to check for the presence and extent of heterogeneity [54]. A meta-analysis using data from all the included studies was not feasible for several reasons including methodological diversity, clinical diversity (e.g. heterogeneity in study participants' age, geographic location, urban or rural settings) and statistical heterogeneity [54]. Performing a meta-analysis in such cases can lead to misleading results [55]. Considering these issues, meta-analyses were only performed for PSAC and pregnant women subgroups. Although the main body of literature identified related to SAC, trying to evaluate a possible association between helminth infection and stunting in this demographic group was complicated by growth changes due to puberty. Most studies in SAC did not take puberty into account, meaning relationships for this group could not be clearly explored.

## Results

**Fig. 1:**
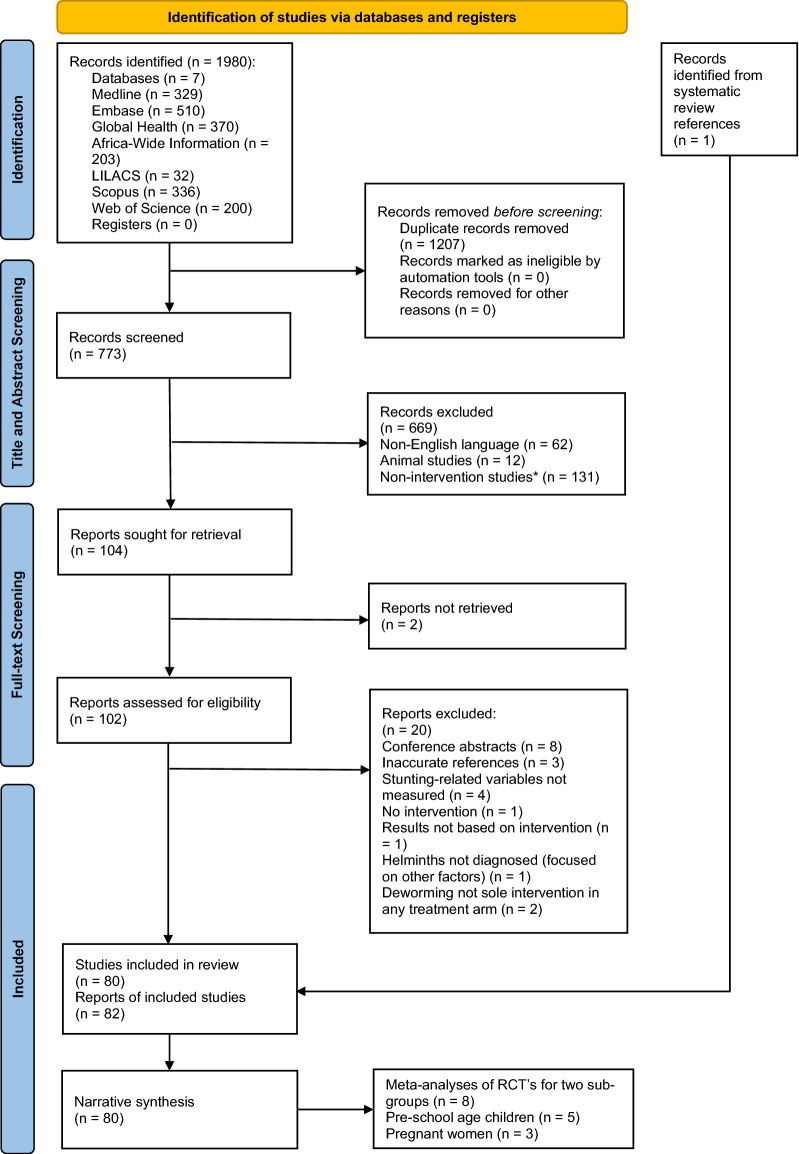
PRISMA flow diagram [48]

Study selection is shown in Fig. 1; a final total of 80 studies were included. The number of studies listed in the “records excluded” box in Fig. 1 does not add up to 669 because the subgroups (e.g. non-English language) underneath represent just a few of the reasons reports were excluded. The 131 non-intervention studies (*) identified at the title and abstract screening stage were excluded because one of the inclusion criteria for this review was anthelmintic treatment, due to the causal nature of the systematic review hypothesis. The discrepancy between the number of reports (*n* = 82) and studies (*n* = 80) is because three reports were on studies already included in the review (*n* = 79), and one additional study was identified from a systematic review reference giving a total of 80 studies. A narrative synthesis was carried out for all included studies and meta-analyses were carried out on RCT studies for two subgroups of PSAC (*n* = 5) and pregnant women (*n* = 3).

A range of different study designs were included in this review, as shown in Table 3, the majority of which were either RCTs (43) or pre-post studies (21).

**Table 3:** Range of study designs included in systematic review

Study design	Number of studies
Case reports	2
Case series	5
Case–control	0
Cluster intervention	1
Community-based non-randomised trial	2
Controlled trial	3
Cross-sectional	1
Pre-post study	21
Randomised controlled trial (RCT)	43
Randomised trial^a^	1
Survey	1
Total	80

^a^[91] Control group deemed unethical, therefore low/moderate/high efficacy intervention groups instead

 (For a table of all included studies sorted by risk of bias, please see Additional file 4.)

Included studies covered several different demographic groups among the population of interest as shown in Table 4. The great majority were on SAC (42), followed by PSAC and SAC (17), infants and PSAC (12), pregnant women (8) and breastfeeding women (1).

**Table 4:** Demographic groups as participants in included studies

Population of interest	Number of studies
Infants and PSAC	12
PSAC and SAC	17
SAC	42
Pregnant women	8
Breastfeeding women	1
Total	80

A range of different helminths were covered, as shown in Table 5; however, most included studies were interested in STHs only (61), and none were identified for FBTs in relation to stunting. Participants in some studies were infected with multiple helminths. However, Table 5 is based on the helminths that were of interest to the researchers and therefore the focus of their studies. For example, for studies listed as STHs only, these may have included participants who were also infected with other helminths such as *Taenia* spp., but the emphasis of the investigations was on STHs only.

**Table 5:** Helminths investigated in included studies

Helminth(s) investigated	Number of studies
STHs only	61
*Schistosoma* spp. only	6
STHs and *Schistosoma* spp.	13
FBTs	0
Total	80

### Case reports and case series

One study [56] included some of the same participants as two other studies [57, 58]. Therefore, to avoid any duplication, the latter two case series were excluded from data synthesis.

Seven eligible case reports and case series were identified in the search results (see Table 6). Although based on a relatively small sample of 10 women, a retrospective case series on pregnant travellers with *Schistosoma* spp. [59] suggested that infected and untreated women can suffer adverse effects such as lower birth weight babies, even many years after exposure. This is noteworthy because women living in endemic areas experience constant helminth exposure. Meanwhile, another case series [56] suggested significant catch-up growth in children following treatment for *Trichuris* dysentery syndrome (TDS), a severe form of helminthiasis. This report emphasised that children could recover height, even after the first 1000 days of life. In another report [60], a 9-month-old baby girl was diagnosed with hookworm infection after concerns were raised regarding growth retardation. Another series [61] reported that all included children were stunted and anaemic; however, they were all hospitalised with TDS. Weight loss was reported in another case report [62], and although poor growth was discussed in relation to TDS, there was no mention of whether the patient was stunted.

**Table 6:** Case reports and case series included in systematic review

Authors	Year	Country	Helminth type	Population of interest	ROB—overall grade	Association found between helminth infection and stunting?
Ben-Chetrit et al.	2015	Israel	schisto	PW	High	Possibly
Callender et al.	1994	Jamaica	STHs	PSAC + SAC	High	Possibly
Cooper et al.	1990	Jamaica	STHs	PSAC + SAC	High	Possibly
Cooper et al.	1995	Jamaica	STHs	PSAC + SAC	High	Possibly
Intra et al.	2019	Italy	STHs	I	High	Possibly
Kaminsky et al.	2015	Honduras	STHs	PSAC + SAC	High	Possibly
Zanwar et al.	2016	India	STHs	SAC	High	Possibly

BFW: breast-feeding women, I: infants (0-1 year old), Possibly: some association between helminth infection and stunting may have been found, or a suggestion of benefit with anthelmintic treatment, but no statistically significant association was found. Also applies when a suspected association was found in case reports and case series, as these study designs provide a weaker level of evidence. (Age group category thresholds for children as defined by WHO [16]), PSAC: pre-school age children (1-5 year olds), PW: pregnant women, ROB: risk of bias, SAC: school age children (5+ to 20 year olds), schisto: schistosomes, STH: soil-transmitted helminths

### Pre-post studies and non-RCTs

Table 7 shows the pre-post and non-RCT studies included in this review. There were 21 pre-post studies, covering both PSAC and SAC, and nine non-RCT studies. Within this group there was significant heterogeneity in terms of sample size, number of faecal and/or urine samples obtained per participant for diagnosis, urban or rural settings, whether treatment was observed and whether an association between helminth infection and stunting was found. Only three studies [63–65] from this group found a significant association between helminth infection and stunting, one of which was in PSAC [65] and another which only found an association for urban children [64]. Out of the remaining nine non-RCT studies, three were in pregnant women [66–68] and two of these found an association between deworming during pregnancy and reduced risk of having a LBW baby [67, 68]. However, none of these studies made use of diagnostic tests to confirm actual helminth infection status and one study [66] used the WHO VLBW threshold to classify babies as LBW, instead of the WHO LBW threshold.

**Table 7:** Pre-post and non-randomised controlled trial studies included in systematic review

Authors	Year	Country	Helminth type	Population of interest	ROB—overall grade	Association found between helminth infection and stunting?
Forrester et al.	1998	Mexico	STHs	PSAC + SAC	Low	Possibly
Ahmed et al.	2012	Malaysia	STHs	SAC	Medium	Yes
Belizario et al.	2014	Philippines	STHs	SAC	Medium	Possibly
Abudho et al.	2020	Kenya	schisto + STHs	SAC	Medium	No
Coutinho et al.	2006	Philippines	schisto	SAC	Medium	Possibly
Degarege et al.	2013	Ethiopia	STHs + schisto	SAC	Medium	No
Efunshile, AM	2017	Nigeria	STHs	PSAC + SAC	Medium	No
Fernando et al.	2001	Sri Lanka	STHs	SAC	Medium	No
Hadju et al.	1998	Indonesia	STHs	SAC	Medium	Possibly
Hagel et al.	1999	Venezuela	STHs	SAC	Medium	Possibly
Halpenny et al.	2013	Panama	STHs	PSAC	Medium	No
Hesham Al-Mekhlafi et al.	2008	Malaysia	STHs	SAC	Medium	No
Humphries et al.	2017	Ghana	STHs	SAC	Medium	No
Hurlimann et al.	2014	Côte d'Ivoire	STHs + schisto	SAC	Medium	No
Kightlinger et al.	1996	Madagascar	STHs	PSAC + SAC	Medium	No
Longfils et al.	2005	Cambodia	STHs	SAC	Medium	No
S Mahendra Raj	1998	Malaysia	STHs	SAC	Medium	No
Osakunor et al.	2018	Zimbabwe	schisto	PSAC	Medium	Yes
Passerini et al.	2012	Vietnam	STHs	PW	Medium	Yes
Shield et al.	1986	Papua New Guinea	STHs	PSAC	Medium	No
Sircar et al.	2018	Kenya	STHs + schisto	SAC	Medium	No
Staudacher et al.	2014	Rwanda	STHs	SAC	Medium	Yes
Stephenson et al.	1980	Kenya	STHs	PSAC + SAC	Medium	No
Stephenson et al.	1989b	Kenya	STHs + schisto	SAC	Medium	No
Tanner et al.	1987	Tanzania	STHs + schisto	PSAC + SAC	Medium	No
Walia et al.	2021	Multi-country	STHs	PW	Medium	Yes
Zhou et al.	2005	China	schisto	SAC	Medium	No
Best et al.	1976	Australia	STHs	SAC	High	No
De Silva et al.	1999	Sri Lanka	STHs	PW	High	Possibly
Echazu et al.	2017	Argentina	STHs	PSAC + SAC	High	Yes

BFW: breast-feeding women, I: infants (0-1 year old), Possibly: some association between helminth infection and stunting may have been found, or a suggestion of benefit with anthelmintic treatment, but no statistically significant association was found. Also applies when a suspected association was found in case reports and case series, as these study designs provide a weaker level of evidence. (Age group category thresholds for children as defined by WHO [16]), PSAC: pre-school age children (1-5 year olds), PW: pregnant women, ROB: risk of bias, SAC: school age children (5+ to 20 year olds), schisto: schistosomes, STH: soil-transmitted helminths

### RCTs

There were 43 RCTs included in this review (see Table 8), covering PSAC, SAC, and pregnant and breastfeeding women. Within this group there was also significant heterogeneity in sample size, blinding (not blinded, single-blind or double-blind), baseline helminth prevalence and anthelmintic treatment. Most studies in this group measured the intensity of helminth infection, and many had follow-up periods of 12 months or more. Only four studies [69–72] from this group found an association between helminth infection and stunting, all of which were in SAC, and three were conducted in sub-Saharan Africa [69, 70, 72]. Although the study in breastfeeding women [73] found no significant difference in infant growth between treatment and placebo groups at 6 months, further analysis of STH-infected mothers revealed significant improvements in infant length gain and length-for-age z-scores (LAZ).

**Table 8:** Randomised controlled trial studies included in systematic review

Authors	Year	Country	Helminth type	Population of interest	ROB—overall grade	Association found between helminth infection and stunting?
Assis et al.	1998	Brazil	schisto	SAC	Low	No
Beach et al.	1999	Haiti	STHs	SAC	Low	No
Greenberg et al.	1981	Bangladesh	STHs	PSAC + SAC	Low	No
Gupta et al.	1982	Guatemala	STHs	PSAC	Low	No
Lai et al.	1995	Malaysia	STHs	SAC	Low	No
Larocque et al.	2006	Peru	STHs	PW	Low	Possibly
Liu et al.	2017	China	STHs	SAC	Low	No
McGarvey et al.	1996	Philippines	schisto	PSAC + SAC	Low	No
KF Michaelsen.	1985	Botswana	STHs	SAC	Low	No
Mofid et al.	2017	Peru	STHs	BFW	Low	Possibly
Ndibazza et al.	2010	Uganda	STHs + schisto	PW	Low	Possibly
Nga et al.	2011	Vietnam	STHs	SAC	Low	No
Northrop-Clewes et al.	2001	Bangladesh	STHs	PSAC	Low	No
Rousham et al.	1994	Bangladesh	STHs	PSAC + SAC	Low	No
Stoltzfus et al.	2004	Tanzania	STHs	PSAC + SAC	Low	No
Taylor et al.	2001	South Africa	STHs + schisto	SAC	Low	No
Watkins & Pollitt	1996	Guatemala	STHs	SAC	Low	No
Willett et al.	1979	Tanzania	STHs	PSAC + SAC	Low	No
Yap et al.	2014	China	STHs	SAC	Low	No
Akpan et al.	2018	Nigeria	STH's	PW	Low	No
Awasthi et al.	2000	India	STHs	PSAC	Medium	No
Befidi-Mengue et al.	1992	Cameroon	schisto + STHs	SAC	Medium	No
Donnen et al.	1998	Democratic Republic of the Congo	STHs	I	Medium	No
Dossa et al.	2001	Benin	STHs	PSAC	Medium	No
Garg et al.	2002	Kenya	STHs	PSAC	Medium	Possibly
Goto et al.	2009	Bangladesh	STHs	I	Medium	No
Hadju et al.	1997	Indonesia	STHs	SAC	Medium	Yes
Hlaing	1994	Myanmar	STHs	PSAC + SAC	Medium	Possibly
Jinabhai et al.	2001a	South Africa	STHs + schisto	SAC	Medium	No
Jinabhai et al.	2001b	South Africa	STHs + schisto	SAC	Medium	No
Joseph et al.	2015	Peru	STHs	I	Medium	No
Kruger et al.	1996	South Africa	STHs	SAC	Medium	No
Nokes et al.	1999	China	STHs + schisto	SAC	Medium	No
Ostwald et al.	1984	Papua New Guinea	STHs	SAC	Medium	No
Sarkar et al.	2002	Bangladesh	STHs	PSAC + SAC	Medium	No
Satya Deepti et al.	2015	India	STHs	PW	Medium	No
Simeon et al.	1995	Jamaica	STHs	SAC	Medium	No
Stephenson et al.	1985	Kenya	STHs + schisto	SAC	Medium	No
Stephenson et al.	1989a	Kenya	STHs	SAC	Medium	Yes
Stephenson et al.	1993	Kenya	STHs	SAC	Medium	Yes
Stoltzfus et al.	1997	Tanzania	STHs	SAC	Medium	Yes
Tee et al.	2013	Malaysia	STHs	SAC	Medium	No
Thein et al.	1991	Myanmar	STHs	SAC	Medium	Possibly

BFW: breast-feeding women, I: infants (0-1 year old), Possibly: some association between helminth infection and stunting may have been found, or a suggestion of benefit with anthelmintic treatment, but no statistically significant association was found. Also applies when a suspected association was found in case reports and case series, as these study designs provide a weaker level of evidence. (Age group category thresholds for children as defined by WHO [16]), PSAC: pre-school age children (1-5 year olds), PW: pregnant women, ROB: risk of bias, SAC: school age children (5+ to 20 year olds), schisto: schistosomes, STH: soil-transmitted helminths

### Meta-analyses

Subgroup meta-analyses were carried out for five RCT studies in PSAC and three RCT studies in pregnant women (two different anthelmintic treatment arms were included for one study). Several RCT studies for PSAC were not included in meta-analyses because of statistically significant differences between treatment and control groups at baseline [74] or data reported were not amenable to meta-analyses [75–77]. One RCT study for pregnant women [78] could not be included in the meta-analysis because the relevant data were not provided separately for treated and control groups.

### Preschool-age children

**Fig. 2:**
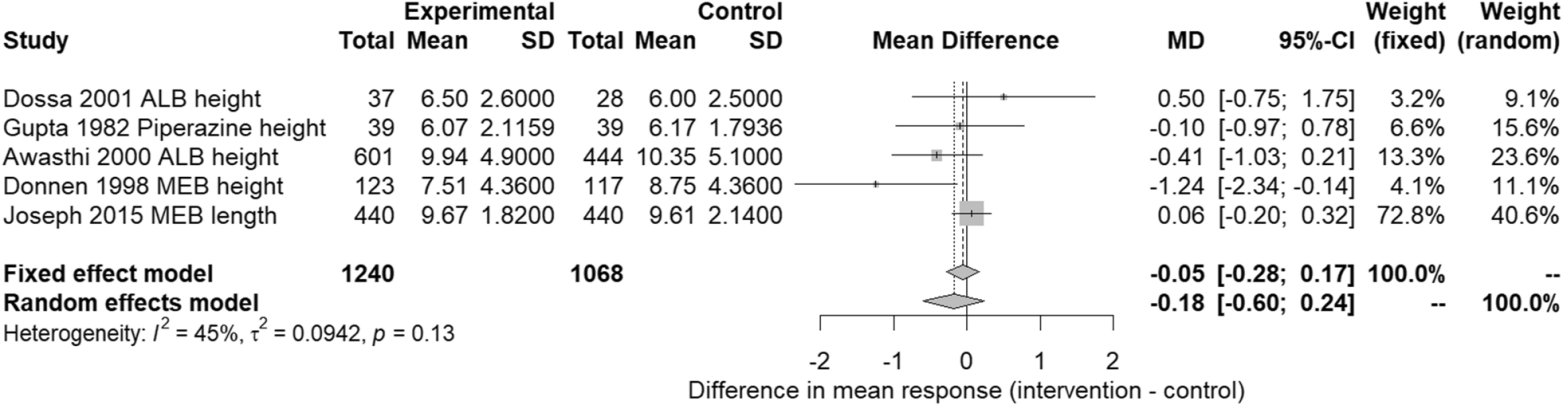
Preschool-age children (PSAC)—effect of anthelmintic treatment on height (cm)

**Fig. 3:**
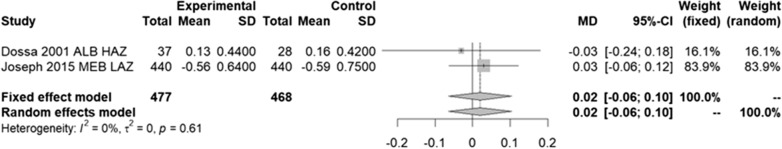
Preschool-age children (PSAC)—effect of anthelmintic treatment on height-for-age/length-for-age z-score

As the meta-analysis in Fig. 2 shows, no significant difference (*p* = 0.40) was found between anthelmintic treatment or placebo on height using either the fixed- or random-effects model. The *I*^2^ statistic detected 45% heterogeneity, indicating moderate but not necessarily important heterogeneity [54]. The meta-analysis results presented in Fig. 3 also showed no significant difference (*p* = 0.63) between anthelmintic treatment or placebo on height-for-age/length-for-age z-scores (HAZ/LAZ) using either the fixed- or random-effects model. The *I*^2^ statistic detected no heterogeneity.

### Pregnant women

**Fig. 4:**
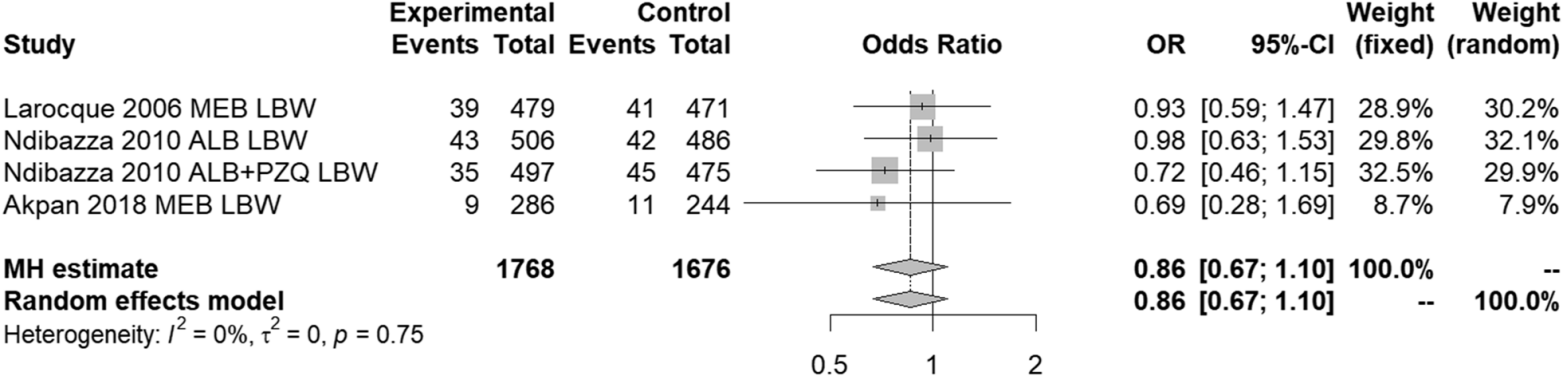
Pregnant women—effect of anthelmintic treatment on low birth weight (< 2500 g)

As Fig. 4 shows, meta-analysis for the pregnant women subgroup found no significant difference (*p* = 0.23) between anthelmintic treatment or placebo on the likelihood of a LBW (< 2500 g) baby using either the fixed- (MH) or random-effects model. The *I*^2^ statistic detected no heterogeneity.

**Fig. 5:**
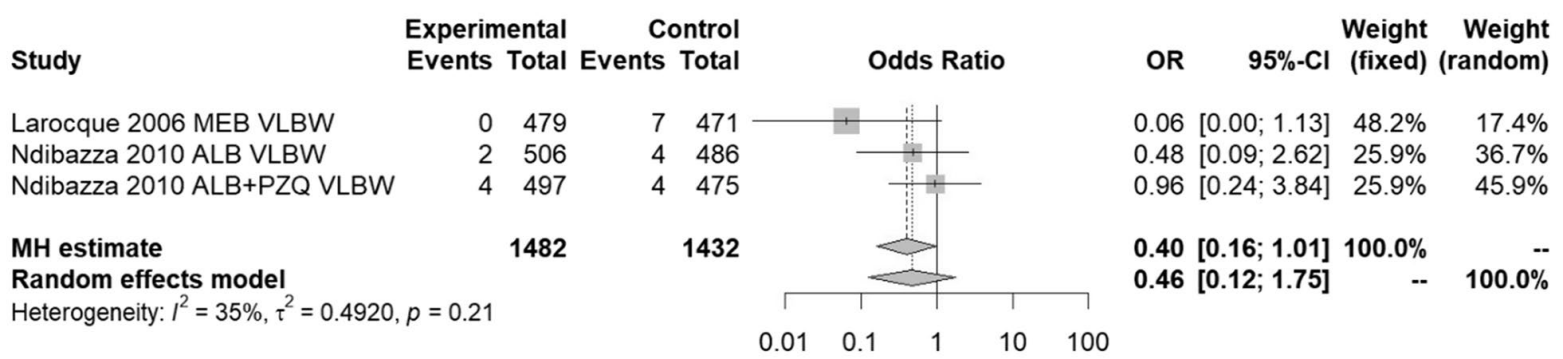
Pregnant women—effect of anthelmintic treatment on very low birth weight (< 1500 g)

The meta-analysis results shown in Fig. 5 for pregnant women also found no significant difference (*p* = 0.26) between anthelmintic treatment or placebo on the likelihood of a VLBW (< 1500 g) baby using either the fixed- (MH) or random-effects model. The *I*^2^ statistic detected 35% heterogeneity, representing moderate but not necessarily important heterogeneity [54].

**Fig. 6:**
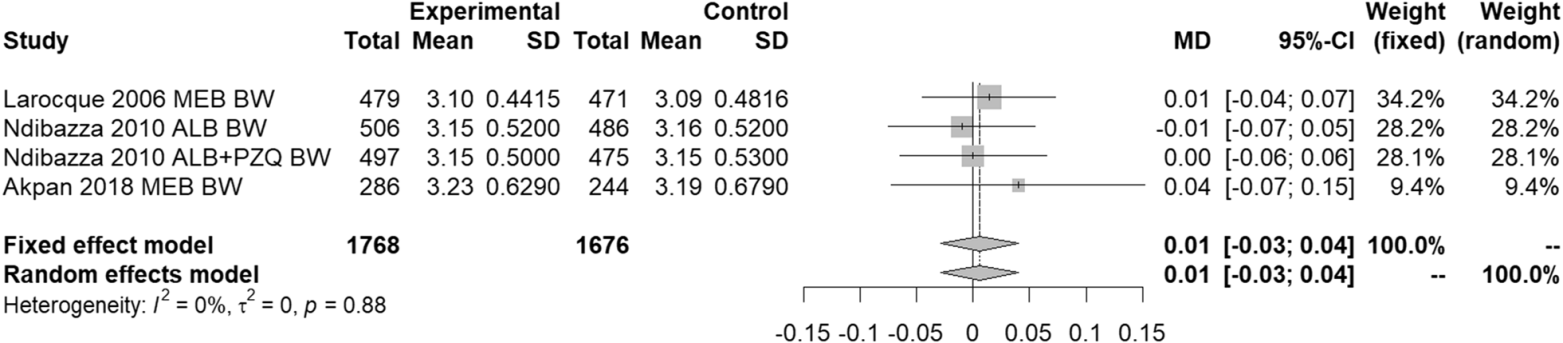
Pregnant women—effect of anthelmintic treatment on mean birth weight (kg)

As Fig. 6 shows, meta-analysis similarly found no significant difference (*p* = 0.74) between anthelmintic treatment or placebo on mean birth weight using either the fixed- or random-effects model. The *I*^2^ statistic detected no heterogeneity.

### Certainty of evidence

Two GRADE summary of findings tables were produced based on the meta-analyses presented; one for PSAC and one for pregnant women [79]. The certainty of the evidence in the PSAC meta-analysis for height was high (Table 9); however, there were serious concerns about risk of bias and possible confounding (see explanations). Similarly, for height-for-age/length-for-age z-scores (HAZ/LAZ), the certainty of the evidence was moderate and there were serious concerns about risk of bias. Table 10 shows the certainty of the evidence in pregnant women was moderate for LBW and mean birth weight, again due to concerns regarding risk of bias (see explanations). The certainty was high for VLBW; however, very few studies were included in that meta-analysis due to limited available data.

**Table 9:** GRADE summary of findings table for preschool-age children (PSAC) [79]

Certainty assessment							No. of patients		Effect		Certainty	Importance
No. of studies	Study design	Risk of bias	Inconsistency	Indirectness	Imprecision	Other considerations	Anthelmintic	Placebo	Relative (95% CI)	Absolute (95% CI)		
Height (cm)												
5	Randomised trials	Serious ^a^	Not serious	Not serious	Not serious	All plausible residual confounding would suggest spurious effect, while no effect was observed	1240	1068	-	MD **0.18 cm fewer** (0.6 fewer to 0.24 more)	⨁⨁⨁⨁ High	CRITICAL
Height-for-age (HAZ) or length-for-age (LAZ)												
2	Randomised trials	Serious ^b^	Not serious	Not serious	Not serious	None	477	468	-	MD **0.02 more** (0.06 fewer to 0.1 more)	⨁⨁⨁◯ Moderate	CRITICAL

Question: Anthelmintic compared to placebo in preschool-age children (PSAC) with helminthiasis to reduce stunting

CI: confidence interval; MD: mean difference

^a^Risk of bias upgraded by one level for height due to insensitive diagnostic test and short follow-up (Dossa et al., 2001), possible confounding due to *Giardia* (Gupta et al., 1982), insensitive diagnostic test and calcium placebo (Awasthi et al., 2000), malnourished children at baseline (Donnen et al., 1998), difference in sensitivity of diagnostic tests used for control and treatment groups and about 25% of participants received treatment from outside of the trial during the trial period (Joseph et al., 2015)

^b^Risk of bias upgraded by one level for HAZ/LAZ due to insensitive diagnostic test and short follow-up (Dossa et al., 2001) and different sensitivity diagnostic tests used for control and treatment groups and about 25% of participants received treatment from outside of the trial during the trial period (Joseph et al., 2015)

GRADE Working Group grades of evidence

High certainty: we are very confident that the true effect lies close to that of the estimate of the effect

Moderate certainty: we are moderately confident in the effect estimate; the true effect is likely to be close to the estimate of the effect, but there is a possibility that it is substantially different

Low certainty: our confidence in the effect estimate is limited; the true effect may be substantially different from the estimate of the effect

Very low certainty: we have very little confidence in the effect estimate; the true effect is likely to be substantially different from the estimate of effect

**Table 10:** GRADE summary of findings table for pregnant women [79]

Certainty assessment							No. of patients		Effect		Certainty	Importance
No. of studies	Study design	Risk of bias	Inconsistency	Indirectness	Imprecision	Other considerations	anthelmintic	placebo	Relative (95% CI)	Absolute (95% CI)		
Low birth weight (< 2500 g)												
4	Randomised trials	Serious ^a^	Not serious	Not serious	Not serious	None	126/1768 (7.1%)	139/1676 (8.3%)	**OR 0.86** (0.67 to 1.10)	**11 fewer per 1000** (from 26 fewer to 8 more)	⨁⨁⨁◯ MODERATE	CRITICAL
Very low birth weight (< 1500 g)												
3	Randomised trials	Not serious	Not serious	Not serious	Not serious	None	6/1482 (0.4%)	15/1432 (1.0%)	**OR 0.46** (0.12 to 1.75)	**6 fewer per 1000** (from 9 fewer to 8 more)	⨁⨁⨁⨁ HIGH	IMPORTANT
Mean birth weight (kg)												
4	Randomised trials	Serious ^b^	Not serious	Not serious	Not serious	None	1768	1676	-	MD **0.01 kg more** (0.03 fewer to 0.04 more)	⨁⨁⨁◯ MODERATE	CRITICAL

CI: confidence interval, MD**:** mean difference, OR: odds ratio, Question: Anthelmintic compared to placebo in pregnant women with helminthiasis to reduce the risk of having a low birth weight baby

^a^Risk of bias was upgraded by one level because one study did not diagnose helminth infection (Akpan et al., 2018)

^b^Risk of bias was upgraded by one level because one study did not diagnose helminth infection (Akpan et al., 2018)

GRADE Working Group grades of evidence

High certainty: we are very confident that the true effect lies close to that of the estimate of the effect

Moderate certainty: we are moderately confident in the effect estimate; the true effect is likely to be close to the estimate of the effect, but there is a possibility that it is substantially different

Low certainty: our confidence in the effect estimate is limited; the true effect may be substantially different from the estimate of the effect

Very low certainty: we have very little confidence in the effect estimate; the true effect is likely to be substantially different from the estimate of effect

## Discussion

The findings of this systematic review indicate, based on the age groups studied to date, there is currently no significant overall evidence that helminths cause physical stunting in children. This is important considering this concept is so frequently cited in the literature and is shown as a cause of stunting in WHO’s stunting conceptual framework [7]. Our findings agree with the most recent Cochrane systematic review on deworming effects in children [41], but contrast with a previous systematic review looking at effects of STHs on child growth and nutrition in settings with > 50% STH prevalence [45] and an empirical analysis of deworming in PSAC based on demographic and health surveys [43]. Notably, however, the present systematic review highlighted the limited available data in the crucial demographic age group of infants and PSAC, as well as pregnant and breastfeeding women. The results also suggested that helminth infection in children may have a greater effect on wasting and/or underweight than on stunting [77, 80–84].

Subgroup meta-analyses for PSAC showed that anthelmintic treatment was not associated with significant improvements in height or HAZ/LAZ (Figs. 2 and 3). However, these meta-analyses were only able to include RCT study designs (to compare treatment with placebo groups); therefore, one non-RCT study in PSAC that did find an association between helminth infection and stunting [65] was not considered. The results of such meta-analyses should therefore be interpreted with caution. Equally, such clear-cut benefit or harm thinking in relation to meta-analyses and GRADE summary of findings tables can lead to more nuanced results being overlooked. For example, one of the included studies in the PSAC meta-analyses [85] did not find an association between helminth infection and stunting but did report a better response in growth when infants were treated at the age of 12 months rather than 18 months. Evidence such as this has the potential to be very useful for clinicians and policymakers, although further similar studies are needed to corroborate such findings. Another study in PSAC [77] reported that although no association was found between helminth infection and stunting, treatment did reduce wasting in children < 30 months old. Again, such information has implications for future research and public health policy, although it would also need verification by other studies.

Importantly, neither the Cochrane Systematic Review nor Hall et al. [41, 45] included, or even considered, the impact of helminthiasis on pregnant women and/or the possible role of stunting beginning in utero. In our study, subgroup meta-analyses showed that deworming during pregnancy did not reduce the risk of having a LBW or VLBW baby, although the evidence base was small and therefore the results must be interpreted with caution. However, as with PSAC, the meta-analyses were only able to include RCT study designs; therefore, non-RCT studies that did find an association between helminth infection and LBW [67, 68] were not considered as part of the analysis. Although not obvious from Fig. 4, one RCT study included in the pregnant women meta-analysis [86] did report a “suggestion of benefit of albendazole among women with moderate to heavy hookworm infection”. Interestingly, the small case series in pregnant travellers [59] found that pregnant women infected with *Schistosoma* spp. who did not receive treatment had babies with lower birth weight compared to those who were treated. Considering that those women were not living in endemic settings and therefore not being constantly re-exposed to helminths, the results are quite remarkable. Given that past reviews [87–89] found no benefit of deworming pregnant women on the outcome of LBW, more, ideally carefully-controlled and multidisciplinary studies are needed to explore this relationship further. One potential mechanism of benefit for pregnant women with helminthiasis who receive anthelmintics is a possible [89] or significant reduction in maternal anaemia [88]. Network meta-analyses [87] that consider anthelmintic treatment for pregnant women in the context of other interventions to improve maternal health may prove more fruitful than evaluating a single intervention at a time.

Pre-post treatment study designs were integral to this review, yet such non-RCT studies tend to be routinely excluded from Cochrane systematic reviews, although the nutritional interventions review is a notable exception [90]. However, for some field intervention studies, a control group is not ethical [63, 91, 92] such as in high helminth prevalence settings or areas where public health programmes are already in place; hence, pre-post treatment study designs are the next best alternative.

When interpreting the results of this review, it is possible that a lack of effect may not necessarily equate to a genuine lack of association between helminth infection and physical stunting in children. This may be because (i) children in the available studies were generally older than the ideal demographic group (infants and PSAC); (ii) follow-up periods may have been too short to see an effect on height (for example, [45] suggests longitudinal studies on growth in children should last 1–2 years); and/or (iii) there may have been low anthelmintic efficacy [93, 94]. For example, as regards the latter point, benzimidazoles such as albendazole and mebendazole, two of the most widely used drugs in STH mass drug administration (MDA) programmes and recommended by WHO [17], have been shown to exhibit reduced efficacy against *Trichuris trichiura* and hookworms [93]. Furthermore, for *S. mansoni*, a study showed potential reduced efficacy of praziquantel in SAC amongst populations that had undergone multiple rounds of MDA [94].

Other factors may also have influenced the findings of studies included in this review. These include differences in available nutrition, helminth prevalence and intensity of infection, helminth species and confounding due to concurrent infections. For example, several studies in children [95, 96] also investigated intestinal protozoa such as *Giardia*, which is known to cause weight loss [97] and has been implicated as a cause of stunting [98, 99]. Other studies [100–102] took place in malaria-endemic regions, which may also have caused confounding, since malaria has been identified as a risk factor for stunting and wasting [103]. Reasons such as these may partly explain the conflicting results of studies that have tried to evaluate whether a potential association exists between helminths and stunting.

This review has demonstrated that significant evidence gaps remain regarding helminths and stunting in children. In summary, we do not yet know whether there is truly no association between helminth infection and physical stunting in children, or whether this is simply due to a scarcity of currently available data for infants and PSAC in their first 1000 days of life. In many cases, we do not know whether a lack of effect on growth is due to reduced anthelmintic efficacy, as this tends not to be routinely monitored [94]. Perhaps most importantly for infants and PSAC, the widely used diagnostic tests such as Kato-Katz and urine filtration are not sufficiently sensitive to detect light infections, which have been shown to be common in this demographic group [104]. This makes it challenging to assess the true disease burden in this important demographic group, and consequently whether any potential association with stunting exists.

As the results highlight, there were few intervention studies that focused on schistosomiasis and stunting (only six, compared with 61 for STHs). This was disappointing, because one of the aims of this review was to evaluate the evidence base for a wide range of helminths in relation to childhood stunting, including schistosomes. Perhaps this demonstrates that stunting is not yet considered a significant indicator of morbidity for schistosomiasis. No intervention studies were found relating to FBTs and potential effects on child growth. This evidence gap should also be urgently addressed, especially as these helminths are now included in the Neglected Tropical Diseases (NTD) 2030 roadmap [105]. Perhaps most starkly of all, despite a comprehensive search strategy, only one intervention study [73] was identified evaluating breastfeeding women with helminthiasis and potential effects on their infant’s growth. This therefore highlights a potentially critical evidence gap.

Some studies included measured the intensity of helminth infection whereas others recorded a binary infected or un-infected result. Assessing the intensity of helminth infections is important because intensity usually correlates with severity of morbidity [16]. It is also possible that potential associations between helminthiasis and stunting only become apparent with higher-intensity infections or in higher-prevalence settings. This too might help explain why one systematic review [45] found an association between helminth infection and stunting, since it only included studies in high-prevalence settings. However, another recent study [106] concluded that treatment to reduce helminth burden might “not improve growth in high *S. mansoni* transmission settings”.

One robust longitudinal cohort study (not eligible for inclusion in this review) found that maternal and child factors were most important for growth during the first two  years, but that environmental factors become more influential in later life [107]. This complements the concept of the nurturing care framework [108], which emphasises the many aspects and layers influencing healthy growth and development of children as they progress through life stages.

It is also important to recognise the limits of stunting as an outcome measure, and to see the condition in the wider context of the social determinants of health, especially in relation to poverty [7, 109, 110]. Researchers, clinicians and policymakers should remain mindful that children can still be short for their age or experiencing growth faltering without necessarily being stunted according to the WHO definition [2]. Undernutrition encompasses stunting, underweight and wasting, yet these are not mutually exclusive conditions, and children may fall into all these categories [6, 111]. Several authors have emphasised the need to address stunting and wasting concurrently rather than treating them as separate issues from a research and policy perspective, since they may share similar risk factors [112, 113]. Some have proposed that physical stunting may partly be the result of previous wasting episodes [113]. Some studies included in our review suggested a possible association of helminthiasis with wasting rather than stunting in children; however, this too needs further exploration since wasting was not the focus of the present study. Considering that helminth infection reduces appetite [114, 115], it might be logical for an association to exist with wasting and/or being underweight. However, this review has also highlighted that much is still unknown, and the evidence base for infants, PSAC, and pregnant and breastfeeding women with helminthiasis is small.

### Limitations

This systematic review had several limitations, the first being that the inclusion and exclusion criteria inevitably led to a degree of publication and language bias. Perhaps the most important limitation was that most of the included studies—by necessity, as that was largely all that was available—focused on SAC. This made evaluating a potential association between helminth infections in children and physical stunting quite challenging, since the first 1000 days are known to be critical for child growth and development [1, 2, 116]. Few of the SAC studies included in our review considered the effect of puberty, which is important because participants in many studies were approaching or in adolescence, and children experience a “pre-adolescent dip” followed by a “pubertal growth spurt” during this time [117]. Evidence suggests that puberty can be delayed by stunting [118], but also that some children who are stunted at preschool-age can experience catch-up growth and recovery from stunting during adolescence [119]. There were relatively few studies on pregnant women (eight) and only one on breastfeeding women, so this is an evidence gap that still needs to be addressed in future research. Some studies also had a relatively short time interval following treatment before anthropometric measurements and faecal and/or urine testing were repeated [120, 121], making changes in height or HAZ more difficult to detect. Many studies used the coprological Kato-Katz diagnostic test, yet more sensitive diagnostic tests, notably those now of multi-parallel quantitative polymerase chain reaction (qPCR), are vital for lower-prevalence settings and lighter-intensity infections [122]. The requirement for RCT study designs for meta-analyses also restricted the number of studies that could be included within them.

### Implications for future research

Studies that combine elements of several of the studies in this review [73, 86] are likely to be most useful for future research, for example deworming during the second or third trimester and then monitoring not only the birth weight of babies, but also their subsequent growth over the first 12 months. Such studies could potentially capture whether helminthiasis leads to intrauterine growth restriction (IUGR) and/or postnatal growth stunting. The benefits of anthelmintic treatment for pregnant women themselves should also not be overlooked. Many women in endemic countries experience several pregnancies during their reproductive lives [32]; therefore, improving their overall health status is likely to have profound repercussions on childhood health. Encouraging countries to record their birth weight data is also essential, as the lack of data currently holds back monitoring and evaluation efforts [123].

The scarcity of available data relating to helminth infections and stunting in the key demographic groups of infants, PSAC, and pregnant and breastfeeding women needs to be urgently addressed. This problem was also clearly highlighted in a recent update of a Cochrane systematic review [89], in which the authors report they were unable to perform planned subgroup analyses due to too few studies. A very recent study [23] looking at early childhood development, stunting and schistosomiasis in Zimbabwe has already begun to address this evidence gap.

Case series such as the study of pregnant travellers infected with schistosomiasis [59] can be very useful. By focusing on travellers from non-endemic regions, that study significantly reduced the chances of confounding, from co-infections for example, which are almost impossible to avoid in endemic areas. Although prone to a high risk of bias, this review shows that case reports and case series can still provide helpful information, and perhaps could be utilised more widely in the scientific literature.

## Conclusion

This systematic review found no overall significant evidence that helminths cause physical stunting in children, albeit some association with wasting and/or undernutrition. However, the main finding was the limited available data for the key demographic groups of infants and PSAC, as well as pregnant and nursing women. Considering the importance of the first 1000 days of life for child growth and development, this evidence gap needs to be urgently addressed in future research. Some of the included studies also suggest there may be a possible relationship between helminth infection and wasting and/or being underweight, indicating that a broader perspective may be needed to evaluate potential relationships between helminth infection and malnutrition in future research.

## Supplementary Information

**Additional file 1.** Study protocol.

**Additional file 2.** Search concepts.

**Additional file 3.** Search Strategies for individual databases.

**Additional file 4.** Table of all included studies.

## Data Availability

PROSPERO record (CRD42021256201) and full data can be made available upon request to the corresponding authors.

## Abbreviations

FBTs: Food-borne trematodes
LBW: Low birth weight
MDA: Mass drug administration
NTD: Neglected tropical diseases
PSAC: Preschool-age children
SAC: School-age children
STHs: Soil-transmitted helminths
VLBW: Very low birth weight
WHO: World Health Organization
