# Supplementary material for: Systematic review to evaluate a potential association between helminth infection and physical stunting in children
Source: Parasit Vectors. 2022 Apr 20;15:135. doi: 10.1186/s13071-022-05235-5 (PMC9022337; doi:10.1186/s13071-022-05235-5)
Supplement: Supplementary file 1 — Additional file 1. Study protocol. [file 13071_2022_5235_MOESM1_ESM.docx]

**S1: Study Protocol**

*Title*

Protocol for a Systematic Review to Evaluate a potential association between Helminth Infection and Childhood Stunting

*Registration*

This systematic review protocol was registered with the International Prospective Register of Systematic Reviews (PROSPERO) on 28/06/21 (registration number; CRD42021256201).

*Background*

Helminths are often cited as a cause of childhood stunting in the literature [1-3], as well as formalised conceptual frameworks such as that of the World Health Organisation (WHO) “Childhood Stunting: Context, Causes and Consequences” [4]. However, the actual role of helminths in physical childhood stunting remains conflicting and unclear. For example, some studies have shown that anthelminthic treatment is associated with reduced stunting in pre-school aged children [5]. Yet others, including an up-dated Cochrane Systematic Review, have reported finding no association between helminthiasis and stunting [6, 7].

Previous systematic reviews looking into this topic have focused on cognitive stunting [8] or have been restricted to soil-transmitted helminths (STHs) only [9]. Others have been based on randomised controlled trials (RCT’s) or quasi-RCT’s only [7], which inevitably miss other relevant studies for which a control group may not have been ethical [10,11]. The current review will cover a range of study designs (not just RCT’s) and focus on physical stunting. It will also include several additional groups of helminths, notably *Strongyloides stercoralis within the STHs*, *Schistosoma* spp. and also key food-borne trematodes (FBTs).

A growing body of evidence shows that physical stunting in children may begin *in utero* [12-15], emphasising the importance of maternal health for early childhood health. Bearing this in mind, this systematic review will also include pregnant and breast-feeding women as participants in relevant studies, rather than only children. Evidence suggests one of the potential causes for low birth weight (<2500g) is intra-uterine growth retardation (IUGR) [15], which has been linked to an increased risk of stunting in childhood [16-18] Aryastami *et al*. 2017). The first 1000 days of life from conception to a child’s second birthday have been widely recognised as a critical window for growth and development [15, 19], therefore this review aims to identify studies relating to infants and pre-school age children in particular.

Considering WHO’s current global targets relating to helminthiasis and stunting, this review is timely and relevant. STHs and schistosomiasis are targeted for elimination as public health problems and FBTs are targeted for control in the recent WHO Neglected Tropical Diseases (NTD) roadmap [20]. Stunting and low birth weight are also targeted for significant reductions as part of the Global Nutrition Targets 2025 [21, 22].

*Specific Objectives*

The objective of this review is to provide evidence in support of or against the hypothesis that helminth infections cause physical stunting in children [23]. The study aims to systematically review relevant studies, in which participants were treated with anthelmintics, and synthesise the evidence to evaluate this potential association. Considering the evidence that stunting can start in utero, pregnant women will also be included in this review. The focus will be on physical stunting, especially in infants and pre-school aged children, therefore the outcomes of interest are expected to be height, height-for-age z-score (HAZ) and low birth weight.

*Eligibility criteria*

**PICO** study characteristics:

**P**opulation and **p**roblem – infants, children, pregnant and breast-feeding women infected with (and/or exposed to) helminths

**I**ntervention – treatment with an anthelmintic

**C**omparison – treated vs controls, infected vs un-infected or pre- and post-treatment

**O**utcome - physical stunting in children (a sub-set of the population of interest)

Inclusion criteria: infection with (and/or exposure to) helminths (STHs, schistosomes or FBTs), participants – children, pregnant or breast-feeding women, anthelmintic treatment intervention, reported stunting-related variables (e.g. height, HAZ, proportion stunted, low birth weight), reported outcomes (helminth infection in participants in relation to stunting), any geographic location, any date, peer-reviewed literature only.

Exclusion criteria; non-primary research (systematic reviews, commentaries and editorials), study protocols, studies with no new or novel data, non-English language papers, animal (non-human) helminth studies.

*Information Sources*

Plan to search seven electronic databases: Medline, Embase, Global Health, Africa-Wide Information, LILACS (Latin American and Caribbean Health Sciences Literature), Scopus and Web of Science. Dates of coverage will be based on individual databases up until the final search date.

*Search strategy*

Draft of search strategy to be used in LILACS database:

helminth$ OR nematod$ OR geohelminth OR "STH" OR schistoso$ OR bilharzia OR ascariasis OR trichuriasis OR ancylostomiasis OR necatoriasis OR "hookworm infection" OR strongyloidiasis OR clonorchiasis OR opisthorchiasis OR fascioliasis OR paragonimiasis OR worm

stunt$ OR "linear growth" OR "growth retardation" OR "growth faltering" OR "growth failure" OR "chronic undernutrition" OR "z-score" OR "height for age" OR "HAZ" OR "height weight" OR anthropometr$ OR preterm OR "premature birth" OR "low birth weight"

child$ OR infant OR toddler OR maternal OR pregnan$ OR "in utero" OR foetal OR fetal OR foetus OR fetus OR lactating OR "breast-feeding" OR neonat$ OR newborn OR paediatric OR pediatric

treatment OR anthelmintic OR "preventive chemotherapy" OR praziquantel OR albendazole OR mebendazole OR ivermectin OR triclabendazole OR deworming OR "mass drug administration" OR "MDA"

*Study records*

Data will be managed in EndNote reference manager.

There will be one main reviewer. Data will be collected by one main reviewer using a piloting form, with another working independently to double-check that appropriate and correct data are extracted. Any discrepancies will be discussed and resolved with help from a third reviewer if required.

Data will be sought relating to prevalence of helminth infection (for example, infected vs un-infected), intensity of infection if present (such as low, moderate or heavy), diagnostics used (for example, Kato-Katz or urine filtration) and time until follow-up. Data will be obtained from studies regarding measurements of height, height/length-for-age, supine length, proportion stunted or low birth weight. Information relating to age of children, location and year of study and treatment intervention will also be sought.

*Risk of Bias in individual studies*

Studies will be assessed for risk of bias by checking for randomisation, comparator groups, adequate blinding and sensitivity and appropriateness of diagnostic tests. The Grading of Recommendations, Assessment, Development and Evaluation (GRADE) framework will also be used to assess risk of bias at the outcome level.

*Data synthesis*

The strategy used will follow the guidelines given in the Cochrane Handbook; chapters 6, 10, 11 and 12 [24-27]. In brief, meta-analysis will be conducted if:

1. studies are homogeneous in terms of participants, interventions and estimated measures of effect
2. estimates and precisions for measures of effect are available, and
3. there is no bias in the evidence provided in the studies

If precision values are not given but can be estimated, these will be derived following guidance given in chapter 6 [24]. If these conditions are not satisfied, then synthesis without meta-analysis (SWiM) [28] will be adopted. Analysis will be carried out in R. The minimum number of studies that will be synthesised is 2. Heterogeneity will be assessed by computing a χ² test and its impact in the analysis will be evaluated by the I² statistic. Sources of heterogeneity will be investigated with subgroup analysis and meta-regression, when appropriate. The measures of effect targeted in relation to categorical variables are risk ratios or odds ratios. However, if other measures of effect are more frequently reported, these will be used instead. In the case of continuous variables, we will look at mean difference, standardised mean difference, or mean ratios. If meta-analysis can be employed, both fixed and random-effects analysis will be conducted, and results reported. The R packages “meta” and “metasens” will be used for meta-analysis. If SWiM is carried out and measures of effect in different studies are available, the range and distribution of observed effects will be synthesised by means of the median and interquartile range. If measures of effect are not reported, then whenever p-values are given, these will be combined to determine whether there is evidence of an effect in at least one of the studies.

*Confidence in cumulative evidence*

The strength of the overall body of evidence will be assessed using Grading of Recommendations, Assessment, Development and Evaluation (GRADE).

References:

1. Crompton DW, Nesheim MC. Nutritional impact of intestinal helminthiasis during the human life cycle. Annu Rev Nutr. 2002;22:35-59.
2. Stephenson LS. Helminth parasites, a major factor in malnutrition. World Health Forum. 1994;15 2:169-72.
3. World Health Organisation. Soil-transmitted helminth infections. Geneva: World Health Organisation, 2020. <https://www.who.int/news-room/fact-sheets/detail/soil-transmitted-helminth-infections>. Accessed 2 May 2021.
4. World Health Organisation. Childhood Stunting Conceptual Framework. Geneva: World Health Organisation, 2013. [https://www.who.int/nutrition/events/2013_ChildhoodStunting_colloquium_14Oct_ConceptualFramework_colour.pdf. Accessed 2 May 2021](https://www.who.int/nutrition/events/2013_ChildhoodStunting_colloquium_14Oct_ConceptualFramework_colour.pdf.%20Accessed%202%20May%202021).
5. Lo NC, Snyder J, Addiss DG, Heft-Neal S, Andrews JR, Bendavid E. Deworming in pre-school age children: A global empirical analysis of health outcomes. PLOS Neglected Tropical Diseases. 2018;12 5:e0006500.
6. Jinabhai CC, Taylor M, Coutsoudis A, Coovadia HM, Tomkins AM, Sullivan KR. Epidemiology of helminth infections: implications for parasite control programmes, a South African perspective. Public Health Nutr. 2001;4 6:1211-9.
7. Taylor‐Robinson DC, Maayan N, Donegan S, Chaplin M, Garner P. Public health deworming programmes for soil‐transmitted helminths in children living in endemic areas. Cochrane Database of Systematic Reviews. 2019; 9.
8. Pabalan N, Singian E, Tabangay L, Jarjanazi H, Boivin MJ, Ezeamama AE. Soil-transmitted helminth infection, loss of education and cognitive impairment in school-aged children: A systematic review and meta-analysis. PLOS Neglected Tropical Diseases. 2018;12 1:e0005523.
9. Hall A, Hewitt G, Tuffrey V, De Silva N. A review and meta-analysis of the impact of intestinal worms on child growth and nutrition. Maternal & Child Nutrition. 2008;4 s1:118-236.
10. Ahmed A, Al-Mekhlafi HM, Al-Adhroey AH, Ithoi I, Abdulsalam AM, Surin J. The nutritional impacts of soil-transmitted helminths infections among Orang Asli schoolchildren in rural Malaysia. Parasit Vectors. 2012;5:119.
11. Crellen T, Sithithaworn P, Pitaksakulrat O, Khuntikeo N, Medley GF, Hollingsworth TD. Towards Evidence-based Control of Opisthorchis viverrini. Trends in Parasitology. 2021;37 5:370-80.
12. Victora CG, de Onis M, Hallal PC, Blössner M, Shrimpton R. Worldwide Timing of Growth Faltering: Revisiting Implications for Interventions. Pediatrics. 2010;125 3:e473.
13. Christian P, Lee SE, Donahue Angel M, Adair LS, Arifeen SE, Ashorn P, et al. Risk of childhood undernutrition related to small-for-gestational age and preterm birth in low- and middle-income countries. Int J Epidemiol. 2013;42 5:1340-55.
14. Prendergast AJ, Rukobo S, Chasekwa B, Mutasa K, Ntozini R, Mbuya MNN, et al. Stunting Is Characterized by Chronic Inflammation in Zimbabwean Infants. PLOS ONE. 2014;9 2:e86928.
15. de Onis M, Branca F. Childhood stunting: a global perspective. Maternal & Child Nutrition. 2016;12 S1:12-26.
16. Sania A, Spiegelman D, Rich-Edwards J, Hertzmark E, Mwiru RS, Kisenge R, et al. The contribution of preterm birth and intrauterine growth restriction to childhood undernutrition in Tanzania. Maternal & Child Nutrition. 2015;11 4:618-30.
17. Blake RA, Park S, Baltazar P, Ayaso EB, Monterde DBS, Acosta LP, et al. LBW and SGA Impact Longitudinal Growth and Nutritional Status of Filipino Infants. PLOS ONE. 2016;11 7:e0159461.
18. Aryastami NK, Shankar A, Kusumawardani N, Besral B, Jahari AB, Achadi E. Low birth weight was the most dominant predictor associated with stunting among children aged 12–23 months in Indonesia. BMC Nutrition. 2017;3 1:16.
19. World Health Organisation. Stunting in a Nutshell. Geneva: World Health Organisation, 2015. <https://www.who.int/news/item/19-11-2015-stunting-in-a-nutshell#:~:text=Stunting%20is%20the%20impaired%20growth,WHO%20Child%20Growth%20Standards%20median>. Accessed 2 May 2021.
20. World Health Organisation. Ending the Neglect to attain the Sustainable Development Goals: A road map for neglected tropical diseases 2021–2030. Geneva: World Health Organisation, 2020. <https://www.who.int/neglected_diseases/Ending-the-neglect-to-attain-the-SDGs--NTD-Roadmap.pdf>. Accessed 2 May 2021.
21. World Health Organisation. Global Nutrition Targets 2025: Stunting Policy Brief, Geneva: World Health Organisation, 2014. <http://apps.who.int/iris/bitstream/handle/10665/149019/WHO_NMH_NHD_14.3_eng.pdf?ua=1>. Accessed 2 May 2021.
22. World Health Organisation. Global Nutrition Targets 2025: Low Birth Weight Policy Brief, Geneva: World Health Organisation, 2014. <https://apps.who.int/iris/handle/10665/149020>. Accessed 2 May 2021.
23. Shamseer L, Moher D, Clarke M, Ghersi D, Liberati A, Petticrew M, et al. Preferred reporting items for systematic review and meta-analysis protocols (PRISMA-P) 2015: elaboration and explanation. BMJ : British Medical Journal. 2015;349:g7647.
24. Higgins, JPT., Li, T., Deeks, J. Chapter 6: Choosing effect measures and computing estimates of effect. In: Higgins JPT, Thomas J, Chandler J, Cumpston M, Li T, Page MJ, Welch VA (editors). 2021. Cochrane Handbook for Systematic Reviews of Interventions version 6.2 (updated February 2021). Cochrane. Available from [www.training.cochrane.org/handbook](http://www.training.cochrane.org/handbook). Accessed 25 June 2021.
25. Altman, D., Ashby, D., Birks, J., Borenstein, M., Campbell, M., Deeks, J., et al. Chapter 10: Analysing Data and undertaking Meta-analyses. In: Higgins JPT, Thomas J, Chandler J, Cumpston M, Li T, Page MJ, Welch VA (editors). 2021. Cochrane Handbook for Systematic Reviews of Interventions version 6.2 (updated February 2021). Cochrane. Available from [www.training.cochrane.org/handbook](http://www.training.cochrane.org/handbook). Accessed 25 June 2021.
26. Chaimani, A., Caldwell, D., Li, T., Higgins, JPT., Salanti, G. Chapter 11: Undertaking network meta-analyses. In: Higgins JPT, Thomas J, Chandler J, Cumpston M, Li T, Page MJ, Welch VA (editors). 2021. Cochrane Handbook for Systematic Reviews of Interventions version 6.2 (updated February 2021). Cochrane. Available from [www.training.cochrane.org/handbook](http://www.training.cochrane.org/handbook). Accessed 25 June 2021.
27. McKenzie, JE., Brennan, SE. Chapter 12: Synthesizing and presenting findings using other methods. In: Higgins JPT, Thomas J, Chandler J, Cumpston M, Li T, Page MJ, Welch VA (editors). 2021. Cochrane Handbook for Systematic Reviews of Interventions version 6.2 (updated February 2021). Cochrane. Available from [www.training.cochrane.org/handbook](http://www.training.cochrane.org/handbook). Accessed 25 June 2021.
28. Campbell M, McKenzie JE, Sowden A, Katikireddi SV, Brennan SE, Ellis S, et al. Synthesis without meta-analysis (SWiM) in systematic reviews: reporting guideline. BMJ. 2020;368:l6890.
