# Supplementary material for: Systematic review to evaluate a potential association between helminth infection and physical stunting in children
Source: Parasit Vectors. 2022 Apr 20;15:135. doi: 10.1186/s13071-022-05235-5 (PMC9022337; doi:10.1186/s13071-022-05235-5)
Supplement: Supplementary file 2 — Additional file 2. Search concepts. [file 13071_2022_5235_MOESM2_ESM.docx]

**Search Concepts**

| ***Concept 1*** | ***Concept 2*** |
| --- | --- |
| helminth* | stunt* |
| nematod* | linear adj3 growth |
| geohelminth? | growth adj3 retardation |
| “STH” | growth adj3 faltering |
| schistoso* | growth adj3 failure |
| Bilharzia | “chronic undernutrition”/ “chronic under-nutrition” |
| Ascariasis | “z-score” |
| Trichuriasis | “height for age” |
| Ancylostomiasis | “HAZ” |
| Necatoriasis | height adj3 weight |
| “hookworm infection?” | anthropometr* |
| Strongyloidiasis | preterm/pre-term |
| Clonorchiasis | “premature birth” |
| Opisthorchiasis | “low birth weight” |
| Fascioliasis |  |
| Paragonimiasis |  |
| Worm |  |

| ***Concept 3*** | ***Concept 4*** |
| --- | --- |
| child* | treatment? |
| infant? | anthelmintic? |
| toddler? | “preventive chemotherapy” |
| Maternal | praziquantel |
| pregnan* | albendazole |
| “in utero” | mebendazole |
| Foetus | ivermectin |
| Fetus | triclabendazole |
| Foetal | deworming/de-worming |
| Fetal | “mass drug administration” |
| Lactating | “MDA” |
| “breast-feeding” |  |
| neonat* |  |
| newborn or new-born |  |
| Paediatric |  |
| Pediatric |  |

Each term within a concept (column) is linked by OR. Then all 4 search concepts are combined using AND in each database.

***Subject Headings – Medline***

*Concept 1* – helminthiasis/, ascariasis/, trichuriasis/, hookworm infections/, schistosomiasis/

*Concept 2 –* body height/, child development/, premature birth/, fetal growth retardation/

*Concept 3* – child/, child, preschool/, infant/, infant, newborn/, pregnancy/, lactation/, breast feeding/, fetus/

*Concept 4* – anthelmintics/

***Subject Headings – Embase***

*Concept 1 –* helminthiasis/, ascariasis/, trichuriasis/, hookworm infection/, schistosomiasis/

*Concept 2 –* stunting/, body height/, prematurity/

*Concept 3 –* child/, infant/, toddler/, newborn/, preschool child/, pregnancy/, breast feeding/, fetus/

*Concept 4 –* antihelminthic therapy/

***Subject Headings – Global Health***

*Concept 1 –* helminthoses/, ascariasis/, trichuriasis/, hookworms/, schistosomiasis/

*Concept 2 –* height/, prematurity/

*Concept 3 –* children/, preschool children/, infants/, neonates/, pregnancy/, breast feeding/, fetus/

*Concept 4 –* anthelmintics/

***Subject Headings – Scopus***

Available but not well used and unreliable. The London School of Hygiene and Tropical Medicine librarians advised against the use of subject headings with this database.

***Subject Headings – LILACS***

The LILACS (Latin American and Caribbean Health Sciences Literature) database uses subject descriptors as an equivalent for medical subject headings, however these are not well used and during trial and error searches, these hindered rather than helped.

***Subject Headings – Africa-Wide Information***

Not available for this database.

***Subject Headings – Web of Science***

Not available for this database.
