# Supplementary material for: Systematic review to evaluate a potential association between helminth infection and physical stunting in children
Source: Parasit Vectors. 2022 Apr 20;15:135. doi: 10.1186/s13071-022-05235-5 (PMC9022337; doi:10.1186/s13071-022-05235-5)
Supplement: Supplementary file 3 — Additional file 3. Search Strategies for individual databases. [file 13071_2022_5235_MOESM3_ESM.docx]

**Search Strategies for each Database**

**Medline:**

| **#** | **Query** |
| --- | --- |
| 1 | helminth*.mp. [mp=title, abstract, original title, name of substance word, subject heading word, floating sub-heading word, keyword heading word, organism supplementary concept word, protocol supplementary concept word, rare disease supplementary concept word, unique identifier, synonyms] |
| 2 | nematod*.mp. [mp=title, abstract, original title, name of substance word, subject heading word, floating sub-heading word, keyword heading word, organism supplementary concept word, protocol supplementary concept word, rare disease supplementary concept word, unique identifier, synonyms] |
| 3 | geohelminth?.mp. [mp=title, abstract, original title, name of substance word, subject heading word, floating sub-heading word, keyword heading word, organism supplementary concept word, protocol supplementary concept word, rare disease supplementary concept word, unique identifier, synonyms] |
| 4 | "STH".mp. [mp=title, abstract, original title, name of substance word, subject heading word, floating sub-heading word, keyword heading word, organism supplementary concept word, protocol supplementary concept word, rare disease supplementary concept word, unique identifier, synonyms] |
| 5 | schistoso*.mp. [mp=title, abstract, original title, name of substance word, subject heading word, floating sub-heading word, keyword heading word, organism supplementary concept word, protocol supplementary concept word, rare disease supplementary concept word, unique identifier, synonyms] |
| 6 | bilharzia.mp. [mp=title, abstract, original title, name of substance word, subject heading word, floating sub-heading word, keyword heading word, organism supplementary concept word, protocol supplementary concept word, rare disease supplementary concept word, unique identifier, synonyms] |
| 7 | ascariasis.mp. [mp=title, abstract, original title, name of substance word, subject heading word, floating sub-heading word, keyword heading word, organism supplementary concept word, protocol supplementary concept word, rare disease supplementary concept word, unique identifier, synonyms] |
| 8 | trichuriasis.mp. [mp=title, abstract, original title, name of substance word, subject heading word, floating sub-heading word, keyword heading word, organism supplementary concept word, protocol supplementary concept word, rare disease supplementary concept word, unique identifier, synonyms] |
| 9 | ancylostomiasis.mp. [mp=title, abstract, original title, name of substance word, subject heading word, floating sub-heading word, keyword heading word, organism supplementary concept word, protocol supplementary concept word, rare disease supplementary concept word, unique identifier, synonyms] |
| 10 | necatoriasis.mp. [mp=title, abstract, original title, name of substance word, subject heading word, floating sub-heading word, keyword heading word, organism supplementary concept word, protocol supplementary concept word, rare disease supplementary concept word, unique identifier, synonyms] |
| 11 | "hookworm infection?".mp. [mp=title, abstract, original title, name of substance word, subject heading word, floating sub-heading word, keyword heading word, organism supplementary concept word, protocol supplementary concept word, rare disease supplementary concept word, unique identifier, synonyms] |
| 12 | strongyloidiasis.mp. [mp=title, abstract, original title, name of substance word, subject heading word, floating sub-heading word, keyword heading word, organism supplementary concept word, protocol supplementary concept word, rare disease supplementary concept word, unique identifier, synonyms] |
| 13 | clonorchiasis.mp. [mp=title, abstract, original title, name of substance word, subject heading word, floating sub-heading word, keyword heading word, organism supplementary concept word, protocol supplementary concept word, rare disease supplementary concept word, unique identifier, synonyms] |
| 14 | opisthorchiasis.mp. [mp=title, abstract, original title, name of substance word, subject heading word, floating sub-heading word, keyword heading word, organism supplementary concept word, protocol supplementary concept word, rare disease supplementary concept word, unique identifier, synonyms] |
| 15 | fascioliasis.mp. [mp=title, abstract, original title, name of substance word, subject heading word, floating sub-heading word, keyword heading word, organism supplementary concept word, protocol supplementary concept word, rare disease supplementary concept word, unique identifier, synonyms] |
| 16 | paragonimiasis.mp. [mp=title, abstract, original title, name of substance word, subject heading word, floating sub-heading word, keyword heading word, organism supplementary concept word, protocol supplementary concept word, rare disease supplementary concept word, unique identifier, synonyms] |
| 17 | helminthiasis/ |
| 18 | ascariasis/ |
| 19 | trichuriasis/ |
| 20 | hookworm infections/ |
| 21 | schistosomiasis/ |
| 22 | 1 or 2 or 3 or 4 or 5 or 6 or 7 or 8 or 9 or 10 or 11 or 12 or 13 or 14 or 15 or 16 or 17 or 18 or 19 or 20 or 21 |
| 23 | stunt*.mp. [mp=title, abstract, original title, name of substance word, subject heading word, floating sub-heading word, keyword heading word, organism supplementary concept word, protocol supplementary concept word, rare disease supplementary concept word, unique identifier, synonyms] |
| 24 | (linear adj3 growth).mp. [mp=title, abstract, original title, name of substance word, subject heading word, floating sub-heading word, keyword heading word, organism supplementary concept word, protocol supplementary concept word, rare disease supplementary concept word, unique identifier, synonyms] |
| 25 | (growth adj3 retardation).mp. [mp=title, abstract, original title, name of substance word, subject heading word, floating sub-heading word, keyword heading word, organism supplementary concept word, protocol supplementary concept word, rare disease supplementary concept word, unique identifier, synonyms] |
| 26 | (growth adj3 faltering).mp. [mp=title, abstract, original title, name of substance word, subject heading word, floating sub-heading word, keyword heading word, organism supplementary concept word, protocol supplementary concept word, rare disease supplementary concept word, unique identifier, synonyms] |
| 27 | (growth adj3 failure).mp. [mp=title, abstract, original title, name of substance word, subject heading word, floating sub-heading word, keyword heading word, organism supplementary concept word, protocol supplementary concept word, rare disease supplementary concept word, unique identifier, synonyms] |
| 28 | "chronic undernutrition".mp. [mp=title, abstract, original title, name of substance word, subject heading word, floating sub-heading word, keyword heading word, organism supplementary concept word, protocol supplementary concept word, rare disease supplementary concept word, unique identifier, synonyms] |
| 29 | "z-score".mp. [mp=title, abstract, original title, name of substance word, subject heading word, floating sub-heading word, keyword heading word, organism supplementary concept word, protocol supplementary concept word, rare disease supplementary concept word, unique identifier, synonyms] |
| 30 | "height for age".mp. [mp=title, abstract, original title, name of substance word, subject heading word, floating sub-heading word, keyword heading word, organism supplementary concept word, protocol supplementary concept word, rare disease supplementary concept word, unique identifier, synonyms] |
| 31 | "HAZ".mp. [mp=title, abstract, original title, name of substance word, subject heading word, floating sub-heading word, keyword heading word, organism supplementary concept word, protocol supplementary concept word, rare disease supplementary concept word, unique identifier, synonyms] |
| 32 | (height adj3 weight).mp. [mp=title, abstract, original title, name of substance word, subject heading word, floating sub-heading word, keyword heading word, organism supplementary concept word, protocol supplementary concept word, rare disease supplementary concept word, unique identifier, synonyms] |
| 33 | anthropometr*.mp. [mp=title, abstract, original title, name of substance word, subject heading word, floating sub-heading word, keyword heading word, organism supplementary concept word, protocol supplementary concept word, rare disease supplementary concept word, unique identifier, synonyms] |
| 34 | preterm.mp. [mp=title, abstract, original title, name of substance word, subject heading word, floating sub-heading word, keyword heading word, organism supplementary concept word, protocol supplementary concept word, rare disease supplementary concept word, unique identifier, synonyms] |
| 35 | "premature birth".mp. [mp=title, abstract, original title, name of substance word, subject heading word, floating sub-heading word, keyword heading word, organism supplementary concept word, protocol supplementary concept word, rare disease supplementary concept word, unique identifier, synonyms] |
| 36 | "low birth weight".mp. [mp=title, abstract, original title, name of substance word, subject heading word, floating sub-heading word, keyword heading word, organism supplementary concept word, protocol supplementary concept word, rare disease supplementary concept word, unique identifier, synonyms] |
| 37 | body height/ |
| 38 | child development/ |
| 39 | premature birth/ |
| 40 | fetal growth retardation/ |
| 41 | 23 or 24 or 25 or 26 or 27 or 28 or 29 or 30 or 31 or 32 or 33 or 34 or 35 or 36 or 37 or 38 or 39 or 40 |
| 42 | child*.mp. [mp=title, abstract, original title, name of substance word, subject heading word, floating sub-heading word, keyword heading word, organism supplementary concept word, protocol supplementary concept word, rare disease supplementary concept word, unique identifier, synonyms] |
| 43 | infant?.mp. [mp=title, abstract, original title, name of substance word, subject heading word, floating sub-heading word, keyword heading word, organism supplementary concept word, protocol supplementary concept word, rare disease supplementary concept word, unique identifier, synonyms] |
| 44 | toddler?.mp. [mp=title, abstract, original title, name of substance word, subject heading word, floating sub-heading word, keyword heading word, organism supplementary concept word, protocol supplementary concept word, rare disease supplementary concept word, unique identifier, synonyms] |
| 45 | maternal.mp. [mp=title, abstract, original title, name of substance word, subject heading word, floating sub-heading word, keyword heading word, organism supplementary concept word, protocol supplementary concept word, rare disease supplementary concept word, unique identifier, synonyms] |
| 46 | pregnan*.mp. [mp=title, abstract, original title, name of substance word, subject heading word, floating sub-heading word, keyword heading word, organism supplementary concept word, protocol supplementary concept word, rare disease supplementary concept word, unique identifier, synonyms] |
| 47 | "in utero".mp. [mp=title, abstract, original title, name of substance word, subject heading word, floating sub-heading word, keyword heading word, organism supplementary concept word, protocol supplementary concept word, rare disease supplementary concept word, unique identifier, synonyms] |
| 48 | foetus.mp. [mp=title, abstract, original title, name of substance word, subject heading word, floating sub-heading word, keyword heading word, organism supplementary concept word, protocol supplementary concept word, rare disease supplementary concept word, unique identifier, synonyms] |
| 49 | fetus.mp. [mp=title, abstract, original title, name of substance word, subject heading word, floating sub-heading word, keyword heading word, organism supplementary concept word, protocol supplementary concept word, rare disease supplementary concept word, unique identifier, synonyms] |
| 50 | foetal.mp. [mp=title, abstract, original title, name of substance word, subject heading word, floating sub-heading word, keyword heading word, organism supplementary concept word, protocol supplementary concept word, rare disease supplementary concept word, unique identifier, synonyms] |
| 51 | fetal.mp. [mp=title, abstract, original title, name of substance word, subject heading word, floating sub-heading word, keyword heading word, organism supplementary concept word, protocol supplementary concept word, rare disease supplementary concept word, unique identifier, synonyms] |
| 52 | lactating.mp. [mp=title, abstract, original title, name of substance word, subject heading word, floating sub-heading word, keyword heading word, organism supplementary concept word, protocol supplementary concept word, rare disease supplementary concept word, unique identifier, synonyms] |
| 53 | "breast-feeding".mp. [mp=title, abstract, original title, name of substance word, subject heading word, floating sub-heading word, keyword heading word, organism supplementary concept word, protocol supplementary concept word, rare disease supplementary concept word, unique identifier, synonyms] |
| 54 | neonat*.mp. [mp=title, abstract, original title, name of substance word, subject heading word, floating sub-heading word, keyword heading word, organism supplementary concept word, protocol supplementary concept word, rare disease supplementary concept word, unique identifier, synonyms] |
| 55 | newborn.mp. [mp=title, abstract, original title, name of substance word, subject heading word, floating sub-heading word, keyword heading word, organism supplementary concept word, protocol supplementary concept word, rare disease supplementary concept word, unique identifier, synonyms] |
| 56 | paediatric.mp. [mp=title, abstract, original title, name of substance word, subject heading word, floating sub-heading word, keyword heading word, organism supplementary concept word, protocol supplementary concept word, rare disease supplementary concept word, unique identifier, synonyms] |
| 57 | pediatric.mp. [mp=title, abstract, original title, name of substance word, subject heading word, floating sub-heading word, keyword heading word, organism supplementary concept word, protocol supplementary concept word, rare disease supplementary concept word, unique identifier, synonyms] |
| 58 | child/ |
| 59 | child, preschool/ |
| 60 | infant/ |
| 61 | infant, newborn/ |
| 62 | pregnancy/ |
| 63 | lactation/ |
| 64 | breastfeeding/ |
| 65 | fetus/ |
| 66 | 42 or 43 or 44 or 45 or 46 or 47 or 48 or 49 or 50 or 51 or 52 or 53 or 54 or 55 or 56 or 57 or 58 or 59 or 60 or 61 or 62 or 63 or 64 or 65 |
| 67 | treatment?.mp. [mp=title, abstract, original title, name of substance word, subject heading word, floating sub-heading word, keyword heading word, organism supplementary concept word, protocol supplementary concept word, rare disease supplementary concept word, unique identifier, synonyms] |
| 68 | anthelmintic?.mp. [mp=title, abstract, original title, name of substance word, subject heading word, floating sub-heading word, keyword heading word, organism supplementary concept word, protocol supplementary concept word, rare disease supplementary concept word, unique identifier, synonyms] |
| 69 | "preventive chemotherapy".mp. [mp=title, abstract, original title, name of substance word, subject heading word, floating sub-heading word, keyword heading word, organism supplementary concept word, protocol supplementary concept word, rare disease supplementary concept word, unique identifier, synonyms] |
| 70 | praziquantel.mp. [mp=title, abstract, original title, name of substance word, subject heading word, floating sub-heading word, keyword heading word, organism supplementary concept word, protocol supplementary concept word, rare disease supplementary concept word, unique identifier, synonyms] |
| 71 | albendazole.mp. [mp=title, abstract, original title, name of substance word, subject heading word, floating sub-heading word, keyword heading word, organism supplementary concept word, protocol supplementary concept word, rare disease supplementary concept word, unique identifier, synonyms] |
| 72 | mebendazole.mp. [mp=title, abstract, original title, name of substance word, subject heading word, floating sub-heading word, keyword heading word, organism supplementary concept word, protocol supplementary concept word, rare disease supplementary concept word, unique identifier, synonyms] |
| 73 | ivermectin.mp. [mp=title, abstract, original title, name of substance word, subject heading word, floating sub-heading word, keyword heading word, organism supplementary concept word, protocol supplementary concept word, rare disease supplementary concept word, unique identifier, synonyms] |
| 74 | triclabendazole.mp. [mp=title, abstract, original title, name of substance word, subject heading word, floating sub-heading word, keyword heading word, organism supplementary concept word, protocol supplementary concept word, rare disease supplementary concept word, unique identifier, synonyms] |
| 75 | deworming.mp. [mp=title, abstract, original title, name of substance word, subject heading word, floating sub-heading word, keyword heading word, organism supplementary concept word, protocol supplementary concept word, rare disease supplementary concept word, unique identifier, synonyms] |
| 76 | "mass drug administration".mp. [mp=title, abstract, original title, name of substance word, subject heading word, floating sub-heading word, keyword heading word, organism supplementary concept word, protocol supplementary concept word, rare disease supplementary concept word, unique identifier, synonyms] |
| 77 | "MDA".mp. [mp=title, abstract, original title, name of substance word, subject heading word, floating sub-heading word, keyword heading word, organism supplementary concept word, protocol supplementary concept word, rare disease supplementary concept word, unique identifier, synonyms] |
| 78 | anthelmintics/ |
| 79 | 67 or 68 or 69 or 70 or 71 or 72 or 73 or 74 or 75 or 76 or 77 or 78 |
| 80 | 22 and 41 and 66 and 79 |

**Embase:**

| **#** | **Query** |
| --- | --- |
| 1 | helminth*.mp. [mp=title, abstract, heading word, drug trade name, original title, device manufacturer, drug manufacturer, device trade name, keyword, floating subheading word, candidate term word] |
| 2 | nematod*.mp. [mp=title, abstract, heading word, drug trade name, original title, device manufacturer, drug manufacturer, device trade name, keyword, floating subheading word, candidate term word] |
| 3 | geohelminth?.mp. [mp=title, abstract, heading word, drug trade name, original title, device manufacturer, drug manufacturer, device trade name, keyword, floating subheading word, candidate term word] |
| 4 | "STH".mp. [mp=title, abstract, heading word, drug trade name, original title, device manufacturer, drug manufacturer, device trade name, keyword, floating subheading word, candidate term word] |
| 5 | schistoso*.mp. [mp=title, abstract, heading word, drug trade name, original title, device manufacturer, drug manufacturer, device trade name, keyword, floating subheading word, candidate term word] |
| 6 | bilharzia.mp. [mp=title, abstract, heading word, drug trade name, original title, device manufacturer, drug manufacturer, device trade name, keyword, floating subheading word, candidate term word] |
| 7 | ascariasis.mp. [mp=title, abstract, heading word, drug trade name, original title, device manufacturer, drug manufacturer, device trade name, keyword, floating subheading word, candidate term word] |
| 8 | trichuriasis.mp. [mp=title, abstract, heading word, drug trade name, original title, device manufacturer, drug manufacturer, device trade name, keyword, floating subheading word, candidate term word] |
| 9 | ancylostomiasis.mp. [mp=title, abstract, heading word, drug trade name, original title, device manufacturer, drug manufacturer, device trade name, keyword, floating subheading word, candidate term word] |
| 10 | necatoriasis.mp. [mp=title, abstract, heading word, drug trade name, original title, device manufacturer, drug manufacturer, device trade name, keyword, floating subheading word, candidate term word] |
| 11 | "hookworm infection?".mp. [mp=title, abstract, heading word, drug trade name, original title, device manufacturer, drug manufacturer, device trade name, keyword, floating subheading word, candidate term word] |
| 12 | strongyloidiasis.mp. [mp=title, abstract, heading word, drug trade name, original title, device manufacturer, drug manufacturer, device trade name, keyword, floating subheading word, candidate term word] |
| 13 | clonorchiasis.mp. [mp=title, abstract, heading word, drug trade name, original title, device manufacturer, drug manufacturer, device trade name, keyword, floating subheading word, candidate term word] |
| 14 | opisthorchiasis.mp. [mp=title, abstract, heading word, drug trade name, original title, device manufacturer, drug manufacturer, device trade name, keyword, floating subheading word, candidate term word] |
| 15 | fascioliasis.mp. [mp=title, abstract, heading word, drug trade name, original title, device manufacturer, drug manufacturer, device trade name, keyword, floating subheading word, candidate term word] |
| 16 | helminthiasis/ |
| 17 | paragonimiasis.mp. [mp=title, abstract, heading word, drug trade name, original title, device manufacturer, drug manufacturer, device trade name, keyword, floating subheading word, candidate term word] |
| 18 | ascariasis/ |
| 19 | trichuriasis/ |
| 20 | hookworm infection/ |
| 21 | schistosomiasis/ |
| 22 | 1 or 2 or 3 or 4 or 5 or 6 or 7 or 8 or 9 or 10 or 11 or 12 or 13 or 14 or 15 or 16 or 17 or 18 or 19 or 20 or 21 |
| 23 | stunt*.mp. [mp=title, abstract, heading word, drug trade name, original title, device manufacturer, drug manufacturer, device trade name, keyword, floating subheading word, candidate term word] |
| 24 | (linear adj3 growth).mp. [mp=title, abstract, heading word, drug trade name, original title, device manufacturer, drug manufacturer, device trade name, keyword, floating subheading word, candidate term word] |
| 25 | (growth adj3 retardation).mp. [mp=title, abstract, heading word, drug trade name, original title, device manufacturer, drug manufacturer, device trade name, keyword, floating subheading word, candidate term word] |
| 26 | (growth adj3 faltering).mp. [mp=title, abstract, heading word, drug trade name, original title, device manufacturer, drug manufacturer, device trade name, keyword, floating subheading word, candidate term word] |
| 27 | (growth adj3 failure).mp. [mp=title, abstract, heading word, drug trade name, original title, device manufacturer, drug manufacturer, device trade name, keyword, floating subheading word, candidate term word] |
| 28 | "chronic undernutrition".mp. [mp=title, abstract, heading word, drug trade name, original title, device manufacturer, drug manufacturer, device trade name, keyword, floating subheading word, candidate term word] |
| 29 | "z-score".mp. [mp=title, abstract, heading word, drug trade name, original title, device manufacturer, drug manufacturer, device trade name, keyword, floating subheading word, candidate term word] |
| 30 | "height for age".mp. [mp=title, abstract, heading word, drug trade name, original title, device manufacturer, drug manufacturer, device trade name, keyword, floating subheading word, candidate term word] |
| 31 | "HAZ".mp. [mp=title, abstract, heading word, drug trade name, original title, device manufacturer, drug manufacturer, device trade name, keyword, floating subheading word, candidate term word] |
| 32 | (height adj3 weight).mp. [mp=title, abstract, heading word, drug trade name, original title, device manufacturer, drug manufacturer, device trade name, keyword, floating subheading word, candidate term word] |
| 33 | anthropometr*.mp. [mp=title, abstract, heading word, drug trade name, original title, device manufacturer, drug manufacturer, device trade name, keyword, floating subheading word, candidate term word] |
| 34 | preterm.mp. [mp=title, abstract, heading word, drug trade name, original title, device manufacturer, drug manufacturer, device trade name, keyword, floating subheading word, candidate term word] |
| 35 | "premature birth".mp. [mp=title, abstract, heading word, drug trade name, original title, device manufacturer, drug manufacturer, device trade name, keyword, floating subheading word, candidate term word] |
| 36 | "low birth weight".mp. [mp=title, abstract, heading word, drug trade name, original title, device manufacturer, drug manufacturer, device trade name, keyword, floating subheading word, candidate term word] |
| 37 | stunting/ |
| 38 | body height/ |
| 39 | prematurity/ |
| 40 | 23 or 24 or 25 or 26 or 27 or 28 or 29 or 30 or 31 or 32 or 33 or 34 or 35 or 36 or 37 or 38 or 39 |
| 41 | child*.mp. [mp=title, abstract, heading word, drug trade name, original title, device manufacturer, drug manufacturer, device trade name, keyword, floating subheading word, candidate term word] |
| 42 | infant?.mp. [mp=title, abstract, heading word, drug trade name, original title, device manufacturer, drug manufacturer, device trade name, keyword, floating subheading word, candidate term word] |
| 43 | toddler?.mp. [mp=title, abstract, heading word, drug trade name, original title, device manufacturer, drug manufacturer, device trade name, keyword, floating subheading word, candidate term word] |
| 44 | maternal.mp. [mp=title, abstract, heading word, drug trade name, original title, device manufacturer, drug manufacturer, device trade name, keyword, floating subheading word, candidate term word] |
| 45 | pregnan*.mp. [mp=title, abstract, heading word, drug trade name, original title, device manufacturer, drug manufacturer, device trade name, keyword, floating subheading word, candidate term word] |
| 46 | "in utero".mp. [mp=title, abstract, heading word, drug trade name, original title, device manufacturer, drug manufacturer, device trade name, keyword, floating subheading word, candidate term word] |
| 47 | foetus.mp. [mp=title, abstract, heading word, drug trade name, original title, device manufacturer, drug manufacturer, device trade name, keyword, floating subheading word, candidate term word] |
| 48 | fetus.mp. [mp=title, abstract, heading word, drug trade name, original title, device manufacturer, drug manufacturer, device trade name, keyword, floating subheading word, candidate term word] |
| 49 | foetal.mp. [mp=title, abstract, heading word, drug trade name, original title, device manufacturer, drug manufacturer, device trade name, keyword, floating subheading word, candidate term word] |
| 50 | fetal.mp. [mp=title, abstract, heading word, drug trade name, original title, device manufacturer, drug manufacturer, device trade name, keyword, floating subheading word, candidate term word] |
| 51 | lactating.mp. [mp=title, abstract, heading word, drug trade name, original title, device manufacturer, drug manufacturer, device trade name, keyword, floating subheading word, candidate term word] |
| 52 | "breast-feeding".mp. [mp=title, abstract, heading word, drug trade name, original title, device manufacturer, drug manufacturer, device trade name, keyword, floating subheading word, candidate term word] |
| 53 | neonat*.mp. [mp=title, abstract, heading word, drug trade name, original title, device manufacturer, drug manufacturer, device trade name, keyword, floating subheading word, candidate term word] |
| 54 | newborn.mp. [mp=title, abstract, heading word, drug trade name, original title, device manufacturer, drug manufacturer, device trade name, keyword, floating subheading word, candidate term word] |
| 55 | paediatric.mp. [mp=title, abstract, heading word, drug trade name, original title, device manufacturer, drug manufacturer, device trade name, keyword, floating subheading word, candidate term word] |
| 56 | pediatric.mp. [mp=title, abstract, heading word, drug trade name, original title, device manufacturer, drug manufacturer, device trade name, keyword, floating subheading word, candidate term word] |
| 57 | child/ |
| 58 | infant/ |
| 59 | toddler/ |
| 60 | newborn/ |
| 61 | preschool child/ |
| 62 | pregnancy/ |
| 63 | breast feeding/ |
| 64 | fetus/ |
| 65 | 41 or 42 or 43 or 44 or 45 or 46 or 47 or 48 or 49 or 50 or 51 or 52 or 53 or 54 or 55 or 56 or 57 or 58 or 59 or 60 or 61 or 62 or 63 or 64 |
| 66 | treatment?.mp. [mp=title, abstract, heading word, drug trade name, original title, device manufacturer, drug manufacturer, device trade name, keyword, floating subheading word, candidate term word] |
| 67 | anthelmintic?.mp. [mp=title, abstract, heading word, drug trade name, original title, device manufacturer, drug manufacturer, device trade name, keyword, floating subheading word, candidate term word] |
| 68 | "preventive chemotherapy".mp. [mp=title, abstract, heading word, drug trade name, original title, device manufacturer, drug manufacturer, device trade name, keyword, floating subheading word, candidate term word] |
| 69 | praziquantel.mp. [mp=title, abstract, heading word, drug trade name, original title, device manufacturer, drug manufacturer, device trade name, keyword, floating subheading word, candidate term word] |
| 70 | albendazole.mp. [mp=title, abstract, heading word, drug trade name, original title, device manufacturer, drug manufacturer, device trade name, keyword, floating subheading word, candidate term word] |
| 71 | mebendazole.mp. [mp=title, abstract, heading word, drug trade name, original title, device manufacturer, drug manufacturer, device trade name, keyword, floating subheading word, candidate term word] |
| 72 | ivermectin.mp. [mp=title, abstract, heading word, drug trade name, original title, device manufacturer, drug manufacturer, device trade name, keyword, floating subheading word, candidate term word] |
| 73 | triclabendazole.mp. [mp=title, abstract, heading word, drug trade name, original title, device manufacturer, drug manufacturer, device trade name, keyword, floating subheading word, candidate term word] |
| 74 | deworming.mp. [mp=title, abstract, heading word, drug trade name, original title, device manufacturer, drug manufacturer, device trade name, keyword, floating subheading word, candidate term word] |
| 75 | "mass drug administration".mp. [mp=title, abstract, heading word, drug trade name, original title, device manufacturer, drug manufacturer, device trade name, keyword, floating subheading word, candidate term word] |
| 76 | "MDA".mp. [mp=title, abstract, heading word, drug trade name, original title, device manufacturer, drug manufacturer, device trade name, keyword, floating subheading word, candidate term word] |
| 77 | antihelminthic therapy/ |
| 78 | 66 or 67 or 68 or 69 or 70 or 71 or 72 or 73 or 74 or 75 or 76 or 77 |
| 79 | 22 and 40 and 65 and 78 |

**Global Health:**

| **#** | **Query** |
| --- | --- |
| 1 | helminth*.mp. [mp=abstract, title, original title, broad terms, heading words, identifiers, cabicodes] |
| 2 | nematod*.mp. [mp=abstract, title, original title, broad terms, heading words, identifiers, cabicodes] |
| 3 | geohelminth?.mp. [mp=abstract, title, original title, broad terms, heading words, identifiers, cabicodes] |
| 4 | "STH".mp. [mp=abstract, title, original title, broad terms, heading words, identifiers, cabicodes] |
| 5 | schistoso*.mp. [mp=abstract, title, original title, broad terms, heading words, identifiers, cabicodes] |
| 6 | bilharzia.mp. [mp=abstract, title, original title, broad terms, heading words, identifiers, cabicodes] |
| 7 | ascariasis.mp. [mp=abstract, title, original title, broad terms, heading words, identifiers, cabicodes] |
| 8 | trichuriasis.mp. [mp=abstract, title, original title, broad terms, heading words, identifiers, cabicodes] |
| 9 | ancylostomiasis.mp. [mp=abstract, title, original title, broad terms, heading words, identifiers, cabicodes] |
| 10 | necatoriasis.mp. [mp=abstract, title, original title, broad terms, heading words, identifiers, cabicodes] |
| 11 | "hookworm infection?".mp. [mp=abstract, title, original title, broad terms, heading words, identifiers, cabicodes] |
| 12 | strongyloidiasis.mp. [mp=abstract, title, original title, broad terms, heading words, identifiers, cabicodes] |
| 13 | clonorchiasis.mp. [mp=abstract, title, original title, broad terms, heading words, identifiers, cabicodes] |
| 14 | opisthorchiasis.mp. [mp=abstract, title, original title, broad terms, heading words, identifiers, cabicodes] |
| 15 | fascioliasis.mp. [mp=abstract, title, original title, broad terms, heading words, identifiers, cabicodes] |
| 16 | paragonimiasis.mp. [mp=abstract, title, original title, broad terms, heading words, identifiers, cabicodes] |
| 17 | helminthoses/ |
| 18 | ascariasis/ |
| 19 | trichuriasis/ |
| 20 | hookworms/ |
| 21 | schistosomiasis/ |
| 22 | 1 or 2 or 3 or 4 or 5 or 6 or 7 or 8 or 9 or 10 or 11 or 12 or 13 or 14 or 15 or 16 or 17 or 18 or 19 or 20 or 21 |
| 23 | stunt*.mp. [mp=abstract, title, original title, broad terms, heading words, identifiers, cabicodes] |
| 24 | (linear adj3 growth).mp. [mp=abstract, title, original title, broad terms, heading words, identifiers, cabicodes] |
| 25 | (growth adj3 retardation).mp. [mp=abstract, title, original title, broad terms, heading words, identifiers, cabicodes] |
| 26 | (growth adj3 faltering).mp. [mp=abstract, title, original title, broad terms, heading words, identifiers, cabicodes] |
| 27 | (growth adj3 failure).mp. [mp=abstract, title, original title, broad terms, heading words, identifiers, cabicodes] |
| 28 | "chronic undernutrition".mp. [mp=abstract, title, original title, broad terms, heading words, identifiers, cabicodes] |
| 29 | "z-score".mp. [mp=abstract, title, original title, broad terms, heading words, identifiers, cabicodes] |
| 30 | "height for age".mp. [mp=abstract, title, original title, broad terms, heading words, identifiers, cabicodes] |
| 31 | "HAZ".mp. [mp=abstract, title, original title, broad terms, heading words, identifiers, cabicodes] |
| 32 | (height adj3 weight).mp. [mp=abstract, title, original title, broad terms, heading words, identifiers, cabicodes] |
| 33 | anthropometr*.mp. [mp=abstract, title, original title, broad terms, heading words, identifiers, cabicodes] |
| 34 | preterm.mp. [mp=abstract, title, original title, broad terms, heading words, identifiers, cabicodes] |
| 35 | "premature birth".mp. [mp=abstract, title, original title, broad terms, heading words, identifiers, cabicodes] |
| 36 | "low birth weight".mp. [mp=abstract, title, original title, broad terms, heading words, identifiers, cabicodes] |
| 37 | height/ |
| 38 | prematurity/ |
| 39 | 23 or 24 or 25 or 26 or 27 or 28 or 29 or 30 or 31 or 32 or 33 or 34 or 35 or 36 or 37 or 38 |
| 40 | child*.mp. [mp=abstract, title, original title, broad terms, heading words, identifiers, cabicodes] |
| 41 | infant?.mp. [mp=abstract, title, original title, broad terms, heading words, identifiers, cabicodes] |
| 42 | toddler?.mp. [mp=abstract, title, original title, broad terms, heading words, identifiers, cabicodes] |
| 43 | maternal.mp. [mp=abstract, title, original title, broad terms, heading words, identifiers, cabicodes] |
| 44 | pregnan*.mp. [mp=abstract, title, original title, broad terms, heading words, identifiers, cabicodes] |
| 45 | "in utero".mp. [mp=abstract, title, original title, broad terms, heading words, identifiers, cabicodes] |
| 46 | foetus.mp. [mp=abstract, title, original title, broad terms, heading words, identifiers, cabicodes] |
| 47 | fetus.mp. [mp=abstract, title, original title, broad terms, heading words, identifiers, cabicodes] |
| 48 | foetal.mp. [mp=abstract, title, original title, broad terms, heading words, identifiers, cabicodes] |
| 49 | fetal.mp. [mp=abstract, title, original title, broad terms, heading words, identifiers, cabicodes] |
| 50 | lactating.mp. [mp=abstract, title, original title, broad terms, heading words, identifiers, cabicodes] |
| 51 | "breast-feeding".mp. [mp=abstract, title, original title, broad terms, heading words, identifiers, cabicodes] |
| 52 | neonat*.mp. [mp=abstract, title, original title, broad terms, heading words, identifiers, cabicodes] |
| 53 | newborn.mp. [mp=abstract, title, original title, broad terms, heading words, identifiers, cabicodes] |
| 54 | paediatric.mp. [mp=abstract, title, original title, broad terms, heading words, identifiers, cabicodes] |
| 55 | pediatric.mp. [mp=abstract, title, original title, broad terms, heading words, identifiers, cabicodes] |
| 56 | children/ |
| 57 | preschool children/ |
| 58 | infants/ |
| 59 | neonates/ |
| 60 | pegnancy/ |
| 61 | breast feeding/ |
| 62 | fetus/ |
| 63 | 40 or 41 or 42 or 43 or 44 or 45 or 46 or 47 or 48 or 49 or 50 or 51 or 52 or 53 or 54 or 55 or 56 or 57 or 58 or 59 or 60 or 61 or 62 |
| 64 | treatment?.mp. [mp=abstract, title, original title, broad terms, heading words, identifiers, cabicodes] |
| 65 | anthelmintic?.mp. [mp=abstract, title, original title, broad terms, heading words, identifiers, cabicodes] |
| 66 | "preventive chemotherapy".mp. [mp=abstract, title, original title, broad terms, heading words, identifiers, cabicodes] |
| 67 | praziquantel.mp. [mp=abstract, title, original title, broad terms, heading words, identifiers, cabicodes] |
| 68 | albendazole.mp. [mp=abstract, title, original title, broad terms, heading words, identifiers, cabicodes] |
| 69 | mebendazole.mp. [mp=abstract, title, original title, broad terms, heading words, identifiers, cabicodes] |
| 70 | ivermectin.mp. [mp=abstract, title, original title, broad terms, heading words, identifiers, cabicodes] |
| 71 | triclabendazole.mp. [mp=abstract, title, original title, broad terms, heading words, identifiers, cabicodes] |
| 72 | deworming.mp. [mp=abstract, title, original title, broad terms, heading words, identifiers, cabicodes] |
| 73 | "mass drug administration".mp. [mp=abstract, title, original title, broad terms, heading words, identifiers, cabicodes] |
| 74 | "MDA".mp. [mp=abstract, title, original title, broad terms, heading words, identifiers, cabicodes] |
| 75 | anthelmintics/ |
| 76 | 64 or 65 or 66 or 67 or 68 or 69 or 70 or 71 or 72 or 73 or 74 or 75 |
| 77 | 22 and 39 and 63 and 76 |

**Africa-Wide Information:**

# Query Limiters/Expanders

S63 S18 AND S33 AND S50 AND S62 Expanders - Apply equivalent subjects

Search modes - Boolean/Phrase Interface - EBSCOhost Research Databases

Search Screen - Advanced Search

Database - Africa-Wide Information Display

S62 S51 OR S52 OR S53 OR S54 OR S55 OR S56 OR S57 OR S58 OR S59 OR S60 OR S61 Expanders - Apply equivalent subjects

Search modes - Boolean/Phrase Interface - EBSCOhost Research Databases

Search Screen - Advanced Search

Database - Africa-Wide Information Display

S61 "MDA" Expanders - Apply equivalent subjects

Search modes - Boolean/Phrase Interface - EBSCOhost Research Databases

Search Screen - Advanced Search

Database - Africa-Wide Information Display

S60 "mass drug administration" Expanders - Apply equivalent subjects

Search modes - Boolean/Phrase Interface - EBSCOhost Research Databases

Search Screen - Advanced Search

Database - Africa-Wide Information Display

S59 deworming Expanders - Apply equivalent subjects

Search modes - Boolean/Phrase Interface - EBSCOhost Research Databases

Search Screen - Advanced Search

Database - Africa-Wide Information Display

S58 triclabendazole Expanders - Apply equivalent subjects

Search modes - Boolean/Phrase Interface - EBSCOhost Research Databases

Search Screen - Advanced Search

Database - Africa-Wide Information Display

S57 ivermectin Expanders - Apply equivalent subjects

Search modes - Boolean/Phrase Interface - EBSCOhost Research Databases

Search Screen - Advanced Search

Database - Africa-Wide Information Display

S56 mebendazole Expanders - Apply equivalent subjects

Search modes - Boolean/Phrase Interface - EBSCOhost Research Databases

Search Screen - Advanced Search

Database - Africa-Wide Information Display

S55 albendazole Expanders - Apply equivalent subjects

Search modes - Boolean/Phrase Interface - EBSCOhost Research Databases

Search Screen - Advanced Search

Database - Africa-Wide Information Display

S54 praziquantel Expanders - Apply equivalent subjects

Search modes - Boolean/Phrase Interface - EBSCOhost Research Databases

Search Screen - Advanced Search

Database - Africa-Wide Information Display

S53 "preventive chemotherapy" Expanders - Apply equivalent subjects

Search modes - Boolean/Phrase Interface - EBSCOhost Research Databases

Search Screen - Advanced Search

Database - Africa-Wide Information Display

S52 anthelmintic Expanders - Apply equivalent subjects

Search modes - Boolean/Phrase Interface - EBSCOhost Research Databases

Search Screen - Advanced Search

Database - Africa-Wide Information Display

S51 treatment Expanders - Apply equivalent subjects

Search modes - Boolean/Phrase Interface - EBSCOhost Research Databases

Search Screen - Advanced Search

Database - Africa-Wide Information Display

S50 S34 OR S35 OR S36 OR S37 OR S38 OR S39 OR S40 OR S41 OR S42 OR S43 OR S44 OR S45 OR S46 OR S47 OR S48 OR S49 Expanders - Apply equivalent subjects

Search modes - Boolean/Phrase Interface - EBSCOhost Research Databases

Search Screen - Advanced Search

Database - Africa-Wide Information Display

S49 pediatric Expanders - Apply equivalent subjects

Search modes - Boolean/Phrase Interface - EBSCOhost Research Databases

Search Screen - Advanced Search

Database - Africa-Wide Information Display

S48 paediatric Expanders - Apply equivalent subjects

Search modes - Boolean/Phrase Interface - EBSCOhost Research Databases

Search Screen - Advanced Search

Database - Africa-Wide Information Display

S47 newborn Expanders - Apply equivalent subjects

Search modes - Boolean/Phrase Interface - EBSCOhost Research Databases

Search Screen - Advanced Search

Database - Africa-Wide Information Display

S46 neonat* Expanders - Apply equivalent subjects

Search modes - Boolean/Phrase Interface - EBSCOhost Research Databases

Search Screen - Advanced Search

Database - Africa-Wide Information Display

S45 "breast-feeding" Expanders - Apply equivalent subjects

Search modes - Boolean/Phrase Interface - EBSCOhost Research Databases

Search Screen - Advanced Search

Database - Africa-Wide Information Display

S44 lactating Expanders - Apply equivalent subjects

Search modes - Boolean/Phrase Interface - EBSCOhost Research Databases

Search Screen - Advanced Search

Database - Africa-Wide Information Display

S43 fetal Expanders - Apply equivalent subjects

Search modes - Boolean/Phrase Interface - EBSCOhost Research Databases

Search Screen - Advanced Search

Database - Africa-Wide Information Display

S42 foetal Expanders - Apply equivalent subjects

Search modes - Boolean/Phrase Interface - EBSCOhost Research Databases

Search Screen - Advanced Search

Database - Africa-Wide Information Display

S41 fetus Expanders - Apply equivalent subjects

Search modes - Boolean/Phrase Interface - EBSCOhost Research Databases

Search Screen - Advanced Search

Database - Africa-Wide Information Display

S40 foetus Expanders - Apply equivalent subjects

Search modes - Boolean/Phrase Interface - EBSCOhost Research Databases

Search Screen - Advanced Search

Database - Africa-Wide Information Display

S39 "in utero" Expanders - Apply equivalent subjects

Search modes - Boolean/Phrase Interface - EBSCOhost Research Databases

Search Screen - Advanced Search

Database - Africa-Wide Information Display

S38 pregnan* Expanders - Apply equivalent subjects

Search modes - Boolean/Phrase Interface - EBSCOhost Research Databases

Search Screen - Advanced Search

Database - Africa-Wide Information Display

S37 maternal Expanders - Apply equivalent subjects

Search modes - Boolean/Phrase Interface - EBSCOhost Research Databases

Search Screen - Advanced Search

Database - Africa-Wide Information Display

S36 toddler Expanders - Apply equivalent subjects

Search modes - Boolean/Phrase Interface - EBSCOhost Research Databases

Search Screen - Advanced Search

Database - Africa-Wide Information Display

S35 infant Expanders - Apply equivalent subjects

Search modes - Boolean/Phrase Interface - EBSCOhost Research Databases

Search Screen - Advanced Search

Database - Africa-Wide Information Display

S34 child* Expanders - Apply equivalent subjects

Search modes - Boolean/Phrase Interface - EBSCOhost Research Databases

Search Screen - Advanced Search

Database - Africa-Wide Information Display

S33 S19 OR S20 OR S21 OR S22 OR S23 OR S24 OR S25 OR S26 OR S27 OR S28 OR S29 OR S30 OR S31 OR S32 Expanders - Apply equivalent subjects

Search modes - Boolean/Phrase Interface - EBSCOhost Research Databases

Search Screen - Advanced Search

Database - Africa-Wide Information Display

S32 "low birth weight" Expanders - Apply equivalent subjects

Search modes - Boolean/Phrase Interface - EBSCOhost Research Databases

Search Screen - Advanced Search

Database - Africa-Wide Information Display

S31 "premature birth" Expanders - Apply equivalent subjects

Search modes - Boolean/Phrase Interface - EBSCOhost Research Databases

Search Screen - Advanced Search

Database - Africa-Wide Information Display

S30 preterm Expanders - Apply equivalent subjects

Search modes - Boolean/Phrase Interface - EBSCOhost Research Databases

Search Screen - Advanced Search

Database - Africa-Wide Information Display

S29 anthropometr* Expanders - Apply equivalent subjects

Search modes - Boolean/Phrase Interface - EBSCOhost Research Databases

Search Screen - Advanced Search

Database - Africa-Wide Information Display

S28 height N3 weight Expanders - Apply equivalent subjects

Search modes - Boolean/Phrase Interface - EBSCOhost Research Databases

Search Screen - Advanced Search

Database - Africa-Wide Information Display

S27 "HAZ" Expanders - Apply equivalent subjects

Search modes - Boolean/Phrase Interface - EBSCOhost Research Databases

Search Screen - Advanced Search

Database - Africa-Wide Information Display

S26 "height for age" Expanders - Apply equivalent subjects

Search modes - Boolean/Phrase Interface - EBSCOhost Research Databases

Search Screen - Advanced Search

Database - Africa-Wide Information Display

S25 "z-score" Expanders - Apply equivalent subjects

Search modes - Boolean/Phrase Interface - EBSCOhost Research Databases

Search Screen - Advanced Search

Database - Africa-Wide Information Display

S24 "chronic undernutrition" Expanders - Apply equivalent subjects

Search modes - Boolean/Phrase Interface - EBSCOhost Research Databases

Search Screen - Advanced Search

Database - Africa-Wide Information Display

S23 growth N3 failure Expanders - Apply equivalent subjects

Search modes - Boolean/Phrase Interface - EBSCOhost Research Databases

Search Screen - Advanced Search

Database - Africa-Wide Information Display

S22 growth N3 faltering Expanders - Apply equivalent subjects

Search modes - Boolean/Phrase Interface - EBSCOhost Research Databases

Search Screen - Advanced Search

Database - Africa-Wide Information Display

S21 growth N3 retardation Expanders - Apply equivalent subjects

Search modes - Boolean/Phrase Interface - EBSCOhost Research Databases

Search Screen - Advanced Search

Database - Africa-Wide Information Display

S20 linear N3 growth Expanders - Apply equivalent subjects

Search modes - Boolean/Phrase Interface - EBSCOhost Research Databases

Search Screen - Advanced Search

Database - Africa-Wide Information Display

S19 stunt* Expanders - Apply equivalent subjects

Search modes - Boolean/Phrase Interface - EBSCOhost Research Databases

Search Screen - Advanced Search

Database - Africa-Wide Information Display

S18 S1 OR S2 OR S3 OR S4 OR S5 OR S6 OR S7 OR S8 OR S9 OR S10 OR S11 OR S12 OR S13 OR S14 OR S15 OR S16 OR S17 Expanders - Apply equivalent subjects

Search modes - Boolean/Phrase Interface - EBSCOhost Research Databases

Search Screen - Advanced Search

Database - Africa-Wide Information Display

S17 worm Expanders - Apply equivalent subjects

Search modes - Boolean/Phrase Interface - EBSCOhost Research Databases

Search Screen - Advanced Search

Database - Africa-Wide Information Display

S16 paragonimiasis Expanders - Apply equivalent subjects

Search modes - Boolean/Phrase Interface - EBSCOhost Research Databases

Search Screen - Advanced Search

Database - Africa-Wide Information Display

S15 fascioliasis Expanders - Apply equivalent subjects

Search modes - Boolean/Phrase Interface - EBSCOhost Research Databases

Search Screen - Advanced Search

Database - Africa-Wide Information Display

S14 opisthorchiasis Expanders - Apply equivalent subjects

Search modes - Boolean/Phrase Interface - EBSCOhost Research Databases

Search Screen - Advanced Search

Database - Africa-Wide Information Display

S13 clonorchiasis Expanders - Apply equivalent subjects

Search modes - Boolean/Phrase Interface - EBSCOhost Research Databases

Search Screen - Advanced Search

Database - Africa-Wide Information Display

S12 strongyloidiasis Expanders - Apply equivalent subjects

Search modes - Boolean/Phrase Interface - EBSCOhost Research Databases

Search Screen - Advanced Search

Database - Africa-Wide Information Display

S11 "hookworm infection" Expanders - Apply equivalent subjects

Search modes - Boolean/Phrase Interface - EBSCOhost Research Databases

Search Screen - Advanced Search

Database - Africa-Wide Information Display

S10 necatoriasis Expanders - Apply equivalent subjects

Search modes - Boolean/Phrase Interface - EBSCOhost Research Databases

Search Screen - Advanced Search

Database - Africa-Wide Information Display

S9 ancylostomiasis Expanders - Apply equivalent subjects

Search modes - Boolean/Phrase Interface - EBSCOhost Research Databases

Search Screen - Advanced Search

Database - Africa-Wide Information Display

S8 trichuriasis Expanders - Apply equivalent subjects

Search modes - Boolean/Phrase Interface - EBSCOhost Research Databases

Search Screen - Advanced Search

Database - Africa-Wide Information Display

S7 ascariasis Expanders - Apply equivalent subjects

Search modes - Boolean/Phrase Interface - EBSCOhost Research Databases

Search Screen - Advanced Search

Database - Africa-Wide Information Display

S6 bilharzia Expanders - Apply equivalent subjects

Search modes - Boolean/Phrase Interface - EBSCOhost Research Databases

Search Screen - Advanced Search

Database - Africa-Wide Information Display

S5 schistoso* Expanders - Apply equivalent subjects

Search modes - Boolean/Phrase Interface - EBSCOhost Research Databases

Search Screen - Advanced Search

Database - Africa-Wide Information Display

S4 “STH” Expanders - Apply equivalent subjects

Search modes - Boolean/Phrase Interface - EBSCOhost Research Databases

Search Screen - Advanced Search

Database - Africa-Wide Information Display

S3 geohelminth Expanders - Apply equivalent subjects

Search modes - Boolean/Phrase Interface - EBSCOhost Research Databases

Search Screen - Advanced Search

Database - Africa-Wide Information Display

S2 nematod* Expanders - Apply equivalent subjects

Search modes - Boolean/Phrase Interface - EBSCOhost Research Databases

Search Screen - Advanced Search

Database - Africa-Wide Information Display

S1 helminth* Expanders - Apply equivalent subjects

Search modes - Boolean/Phrase Interface - EBSCOhost Research Databases

Search Screen - Advanced Search

Database - Africa-Wide Information Display

**LILACS (Latin American and Caribbean Health Sciences Literature):**

helminth$ OR nematod$ OR geohelminth OR "STH" OR schistoso$ OR bilharzia OR ascariasis OR trichuriasis OR ancylostomiasis OR necatoriasis OR "hookworm infection" OR strongyloidiasis OR clonorchiasis OR opisthorchiasis OR fascioliasis OR paragonimiasis OR worm

stunt$ OR "linear growth" OR "growth retardation" OR "growth faltering" OR "growth failure" OR "chronic undernutrition" OR "z-score" OR "height for age" OR "HAZ" OR "height weight" OR anthropometr$ OR preterm OR "premature birth" OR "low birth weight"

child$ OR infant OR toddler OR maternal OR pregnan$ OR "in utero" OR foetal OR fetal OR foetus OR fetus OR lactating OR "breast-feeding" OR neonat$ OR newborn OR paediatric OR pediatric

treatment OR anthelmintic OR "preventive chemotherapy" OR praziquantel OR albendazole OR mebendazole OR ivermectin OR triclabendazole OR deworming OR "mass drug administration" OR "MDA"

**Scopus:**

( TITLE-ABS-KEY ( helminth* OR nematod* OR geohelminth OR "STH" OR schistoso* OR bilharzia OR ascariasis OR trichuriasis OR ancylostomiasis OR necatoriasis OR "hookworm infection" OR strongyloidiasis OR clonorchiasis OR opisthorchiasis OR fascioliasis OR paragonimiasis OR worm ) ) AND ( TITLE-ABS-KEY ( stunt* OR "linear W/3 growth" OR "growth W/3 retardation" OR "growth W/3 faltering" OR "growth W/3 failure" OR "chronic undernutrition" OR "z-score" OR "height for age" OR "HAZ" OR "height W/3 weight" OR anthropometr* OR preterm OR "premature birth" OR "low birth weight" ) ) AND ( TITLE-ABS-KEY ( child* OR infant OR toddler OR maternal OR pregnan* OR "in utero" OR foetal OR fetal OR foetus OR fetus OR lactating OR "breast-feeding" OR neonat* OR newborn OR paediatric OR pediatric ) ) AND ( TITLE-ABS-KEY ( treatment OR anthelmintic OR "preventive chemotherapy" OR praziquantel OR albendazole OR mebendazole OR ivermectin OR triclabendazole OR deworming OR "mass drug administration" OR "MDA" ) )

**Web of Science:**

(helminth* OR nematod* OR geohelminth OR "STH" OR schistoso* OR bilharzia OR ascariasis OR trichuriasis OR ancylostomiasis OR necatoriasis OR "hookworm infection" OR strongyloidiasis OR clonorchiasis OR opisthorchiasis OR fascioliasis OR paragonimiasis OR worm)

AND

(stunt* OR "linear NEAR/3 growth" OR "growth NEAR/3 retardation" OR "growth NEAR/3 faltering" OR "growth NEAR/3 failure" OR "chronic undernutrition" OR "z-score" OR "height for age" OR "HAZ" OR "height NEAR/3 weight" OR anthropometr* OR preterm OR "premature birth" OR "low birth weight")

AND

(child* OR infant OR toddler OR maternal OR pregnan* OR "in utero" OR foetal OR fetal OR foetus OR fetus OR lactating OR "breast-feeding" OR neonat* OR newborn OR paediatric OR pediatric)

AND

(treatment OR anthelmintic OR "preventive chemotherapy" OR praziquantel OR albendazole OR mebendazole OR ivermectin OR triclabendazole OR deworming OR "mass drug administration" OR "MDA")
